# The control of seed oil polyunsaturate content in the polyploid crop species *Brassica napus*

**DOI:** 10.1007/s11032-013-9954-5

**Published:** 2013-09-21

**Authors:** Rachel Wells, Martin Trick, Eleni Soumpourou, Leah Clissold, Colin Morgan, Peter Werner, Carl Gibbard, Matthew Clarke, Richard Jennaway, Ian Bancroft

**Affiliations:** 1 John Innes Centre, Norwich Research Park, Norwich, NR4 7UH UK; 2 Present Address: The Genome Analysis Centre, Norwich Research Park, Norwich, NR4 7UH UK; 3 KWS UK Ltd., 56 Church Street, Thriplow, Hertfordshire, SG8 7RE UK; 4 Monsanto UK Ltd., PO Box 663, Cambridge, CB1 0LD UK; 5 Saaten-Union UK Ltd., Rosalie Field Station, Bradley Road, Cowlinge, Newmarket, Suffolk, CB8 9HU UK; 6 Present Address: Department of Biology, University of York, Heslington, York, YO41 5DD UK

**Keywords:** Desaturation, Fatty acid biosynthesis, *Brassica*, Oleic acid, Polyunsaturated fatty acids, EMS

## Abstract

Many important plant species have polyploidy in their recent ancestry, complicating inferences about the genetic basis of trait variation. Although the principal locus controlling the proportion of polyunsaturated fatty acids (PUFAs) in seeds of *Arabidopsis thaliana* is known (*fatty acid desaturase* 2; *FAD2*), commercial cultivars of a related crop, oilseed rape (*Brassica napus*), with very low PUFA content have yet to be developed. We showed that a cultivar of oilseed rape with lower than usual PUFA content has non-functional alleles at three of the four orthologous *FAD2* loci. To explore the genetic basis further, we developed an ethyl methanesulphonate mutagenised population, JBnaCAB_E, and used it to identify lines that also carried mutations in the remaining functional copy. This confirmed the hypothesised basis of variation, resulting in an allelic series of mutant lines showing a spectrum of PUFA contents of seed oil. Several lines had PUFA content of ~6 % and oleic acid content of ~84 %, achieving a long-standing industry objective: very high oleic, very low PUFA rapeseed without the use of genetic modification technology. The population contains a high rate of mutations and represents an important resource for research in *B. napus*.

**Electronic supplementary material:**

The online version of this article (doi:10.1007/s11032-013-9954-5) contains supplementary material, which is available to authorized users.

## Introduction

Vegetable oils are an important part of the human diet, providing essential fatty acids. One of the most important oilseed crops, second in global production only to soybean, is oilseed rape (Canola). Oilseed rape is one of the crop types of the species *Brassica napus*. Like *Arabidopsis thaliana*, *B. napus* is a member of the Brassicacea, but unlike the simple genome of *A. thaliana*, that of *B. napus* is polyploid, which is more typical of crop species. The genetic complexity arising from polyploidy presents a barrier for the translation of knowledge from fundamental research in species such as *A. thaliana* into crop improvement, necessitating study of the genetic basis of traits in the crop species themselves. The lack of progress towards long-standing industry objectives in oilseed rape by traditional breeding, such as reducing the content of polyunsaturated fatty acids in rapeseed oil in order to improve its thermal stability, is a good example.

Fatty acid biosynthesis has been studied extensively in *A.* *thaliana*. The major fatty acids in seed oil are derived from the saturated fatty acid moiety stearic (which contains a backbone of 18 carbon atoms with no double bonds and is denoted C18:0). This can be elongated successively to 20 and 24 carbon fatty acids, but in modern rapeseed cultivars that pathway has been blocked by mutation of orthologues of components of the elongase complex encoded by the *FAE1* locus (James et al. 1995), resulting in rapeseed oil comprising predominantly 18 carbon fatty acids. In both *A. thaliana* and rapeseed, C18:0 is sequentially desaturated to the monounsaturated fatty acid (MUFA) oleic (one double bond; denoted C18:1), then the polyunsaturated fatty acid (PUFA) linoleic (two double bonds; denoted C18:2) and finally the PUFA linolenic (three double bonds; denoted C18:3). The key control point in the biosynthesis of PUFAs in *A. thaliana* is the desaturase encoded by the *fatty acid desaturase* 2 (*FAD2*) locus, which catalyses the desaturation of C18:1 to C18:2 (Miquel and Browse 1992). Okuley et al. (1994) reported two T-DNA insertion lines of *A. thaliana* where disruption of the gene resulted in a considerable increase in C18:1 from 15.4 % in the wild type to 37.7 and 53.5 % in the *fad2*-*5* and *fad2*-*1* alleles respectively. This was accompanied by a reduction in C18 PUFA from 53 to 19.4 and 8.5 % respectively, showing that inactivation of the enzyme encoded by the *FAD2* locus (i.e. blocking of the desaturation pathway) results in a high oleic, low PUFA oil profile. In double mutants, in which the biosynthesis of erucic acid (C22:1) is also blocked, C18:1 accumulated to around 85 % in *A. thaliana* (Smith et al. 2003).

The *Brassica* species are the closest crop relatives of the model plant *A. thaliana*. *B. napus* itself is a recently-formed allotetraploid resulting from hybridisation between the “diploid” Brassica species, *B. rapa* (A genome) and *B. oleracea* (C genome). Comparative mapping with *A. thaliana* suggest that the diploid *Brassica* genomes are themselves derived from a hexaploid ancestor, and the set of six related genome segments (three from *B. rapa* and three from *B. oleracea*) can be discerned clearly in *B. napus* (Rana et al. 2004). However, extensive interspersed gene loss has occurred during the diploidisation process following polyploidy (O’Neill and Bancroft 2000; Town et al. 2006; Yang et al. 2006; Cheung et al. 2009), so families of genes in *B. napus* are not necessarily present in six copies. Indeed, recent characterisation of the family of *FAD2* orthologues in *B. napus* (denoted *BnaFAD2*) by Yang et al. (2012a) has confirmed that four are present within *B. napus,* as suggested previously by Scheffler et al. (1997). Family member *BnaA.FAD2.b* contains deletion and insertion events in its coding region, leading to mis-sense mutation and truncation, so is unlikely to be functional. Alleles of *BnaA.FAD2.a* that contain a 4-bp insertion in its coding region, which is expected to abolish function, resulted in an increase in C18:1 content (from 64.5 to 75 % of total fatty acids). The two copies in the C genome (*BnaC.FAD2.a* and *BnaC.FAD2.b*) appear intact and likely to be functional. An approximation of the knock-out of the complete *FAD2* gene family has been achieved by RNAi (Peng et al. 2010), which resulted in an oleic acid content of up to 85 % and a reduction in PUFA to under 10 %. However, the stability, efficacy and precision of RNAi is unclear, particularly in polyploids. The approach is unattractive for crop improvement due to the high cost of completing the regulatory processes, but the results provide encouragement that a clearer understanding of the way in which the *BnaFAD2* gene family controls the trait may enable the predictive breeding of rapeseed producing very low PUFA oil.

It is generally considered that genetic diversity in *B. napus* is low, as the species arose from a limited number of spontaneous hybridisation events in a restricted geographic range (Mei et al. 2011), with the elite rapeseed genepool further eroded by intensive breeding with an emphasis on specific oil and seed quality traits (Hasan et al. 2006). To help overcome a lack of natural genetic variation, induced genetic variation (mutation breeding) techniques have been developed. These include the use of ionising radiation, such as X-rays or gamma rays, and chemical mutagens. Indeed, it has been estimated that over 2,000 varieties resulting from mutation breeding have been released in the last 75 years (FAO/IAEA 2010). Whilst irradiation techniques produce larger-scale genome deletions and rearrangements, chemical mutagenesis induces point mutations which may enable the identification of an allelic series of mutations. EMS produces mutations in genetic material by nucleotide substitution. The principal mechanism is via alkylation of guanine to form O^6^-ethylguanine, which cannot pair with cytosine but can pair with thymine. During subsequent replication, the effect is to substitute thymine for cytosine. Hence the predominant result of EMS mutagenesis is C/G to T/A transition changes, although occasionally G/C to C/G or G/C to T/A transversions, or A/T to G/C transitions occur (Krieg 1963; Greene et al. 2003). EMS has been used successfully for the development of mutagenised *Brassica* populations (Wang et al. 2008; Stephenson et al. 2010; Harloff et al. 2012; Himelblau et al. 2009).

Current approaches to detecting sequence variation in mutation/germplasm screens rely on methods such as Targeting Induced Local Lesions IN Genomes (TILLING) (McCallum et al. 2000; Stemple 2004) or conventional Sanger sequencing of a target amplicon. In TILLING, PCR is used to amplify an exon fragment from the target gene using pooled DNA from the individuals in a mutagenised population. The products are then melted and re-annealed before digestion with Cel1 exonuclease, which cleaves at mismatched bases in heteroduplex DNA (Oleykowski et al. 1998). The PCR fragments are then analysed using fluoro-labelled electrophoresis and mutations confirmed via PCR and sequencing from individual DNA samples. A general requirement for mutation detection involving heteroduplex analysis is the ability to develop a locus-specific PCR amplicon, which can be problematic in polyploid species, although occasionally gene families are so similar in sequence that TILLING can be conducted even with mixed amplicons (Wang et al. 2008).

Our aim was to understand the genetic basis of the control of PUFA content in rapeseed oil by functional characterisation of the family of *FAD2* orthologues in *B. napus*. We based our study on a conventional winter oilseed rape cultivar, Tapidor (oil profile: ~60 % C18:1, 29 % PUFAs), and a cultivar showing reduced PUFA content, Cabriolet (oil profile: ~75 % C18:1, 16 % PUFAs). To test a hypothesis, we developed a new EMS mutagenised population of *B. napus* (JBnaCAB_E) from the latter and analysed the phenotypic effects in an allelic series of mutations in a specific orthologue of *FAD2*.

## Materials and methods

### Determination of homologue number and sequence of *FAD2* homologues within *B. napus* var. Tapidor

Clones from the *B. napus* var. Tapidor JBnY and JBnB Bacterial artificial chromosome (BAC) libraries containing the *BnaFAD2* genes were previously identified as described by Smooker et al. (2011). DNA from all positively hybridising clones confirmed by Southern hybridisation was prepared by standard methods (Marra et al. 1997). Alignments of the *AtFAD2* sequence and *BnaFAD2* sequences downloaded from Genbank produced using VectorNTi AlignX (Invitrogen) (Lu and Moriyama 2004) were used to design a degenerate forward PCR primer (ATTCCTTCCTNCTNCTNGTNCC) and a reverse primer (CAGGAGAAGTAAGGGACGAGG) within a conserved region of the gene. PCR was performed using 1 μl DNA prep, 2 μl 10 × PCR buffer [500 mM KCl, 100 mM Tris–HCl (pH 9.0), 1 % Triton X-100, 15 mM MgCl_2_ (supplied with AmplitaqTaq Gold)], 2 μl forward primer (2 mM), 2 μl reverse primer (2 mM), 1.3 μl dNTPs (2 μM Invitrogen Cat. No. 10297-018), 0.2 μl Amplitaq Gold 5 u/μl (Applied Biosystems Cat. No. 4311820) and 11.5 μl ddH_2_O on the following touchdown cycle: 94 °C for 5 min, 15 cycles of 94 °C for 30 s, 64 °C for 30 s (–1 °C per cycle) and 72 °C for 30 s, 30 cycles of 94 °C for 30 s, 53 °C for 30 s and 72 °C for 30 s, then 72 °C for 7 min, and store at 8 °C. To determine the individual homologues of *FAD2* present within the clones, sequencing was performed on 1 μl of PCR product cleaned up by isopropanol precipitation using the BigDye Terminator v3.1 cycle sequencing kit (Applied Biosystems Cat. No. 100 reactions 4337455) according to the manufacturer’s instructions (Applied Biosystems). Individual clone sequences were again compared using AlignX and divided into homologue groups. To obtain the sequence of the complete *BnaFAD2* homologues, one clone per group was chosen and sequenced by external commercial service providers (JIC Genome Laboratory; GATC-Biotech in Konstanz, Germany; Beijing Genomics Institute). To confirm that no further homologues of *BnFAD2* were present within *B. napus*, cloning of PCR product from a conserved forward primer (CCTCGTCCCTTACTTCTCCTG) and the reverse primer previously used to screen the BAC clones amplified on a standard 50 °C PCR cycle, was performed on genomic Tapidor DNA using the pGEM-T Easy vector system kit according to the manufacturer’s instructions (Promega Cat. No. A1360). Colony PCR and sequencing using the vector SP6 and T7 primers was then performed for 11 clones and sequences aligned against the previously identified homologues.

### Mapping of *BnaFAD2* homologues

Three of the *BnaFAD2* homologues were mapped as previously described by Smooker et al. (2011). The chromosome allocation of the remaining homologue was implied by homology to the *B. rapa* genome sequence (Wang et al. 2011).

### Determination of sequence of *BnaFAD2* homologues within *B. napus* var. Cabriolet

A combination of specific and conserved primers designed from the Tapidor reference sequence was used to obtain the gene sequence from Cabriolet following the touchdown PCR protocol described above, with the first touchdown cycle starting at 63 °C. Primers are detailed in Supplemental Table 1. Primer combinations for amplifying ~1,000 bp of each homologue are given in Supplemental Table 2.

### Expression analysis of *BnaFAD2* homologues

Ten seeds of Tapidor and Cabriolet were sown, pricked out into individual 6-cm pots at the two-leaf stage and, at the four-leaf stage, vernalised at 4 °C for 6 weeks. Following vernalisation, plants were transferred to a glasshouse at 12–18 °C with 16-h day length, and re-potted into 1-L pots. RNA was extracted from developing seed 45 DPA (a stage at which *Bna.FAD2* gene expression is expected to be high) using the RNeasy plant mini kit (Qiagen). Buffer RLC (containing guanidine hydrochloride) was substituted for buffer RLT due to the secondary metabolites present within the seed. cDNA was synthesised using the Superscript III First Strand Synthesis System for RT-PCR (Invitrogen Cat. No. 18080-051). PCR and cloning of RT-PCR product was performed as for the genomic samples, detailed previously.

### Transcriptome sequencing of Tapidor and Cabriolet seed

As amplification could not be achieved for homologue *BnaC.FAD2.a* within Cabriolet, transcriptome sequencing, which does not rely on homologue amplification, was performed on the RNA from developing seed 45 days after pollination (DAP) using the 80-bp Illumina RNA-seq GAII platform by TGAC. Reads were aligned against the Tapidor reference sequences and data viewed using Tablet Next Generation Sequence Assembly Visualisation software (Milne et al. 2010).

### Mutagenesis of *B. napus* var. Cabriolet

EMS mutagenesis was carried out on ~33,000 seeds of *B. napus* var. Cabriolet. Five treatments of 150 ml of 0.2, 0.4, 0.6, 0.8 and 1 % EMS (Sigma-Aldrich Cat. No. M0880) in 0.02 % Tween 20 solution (Sigma-Aldrich Cat. No. TS700-500ML) were performed on 30 g (~6,400) seed each. Treatments of 10 ml 0.02 % Tween 20 and 10 ml 2 % EMS were performed on 2.5 g (~500) seed as negative and positive controls respectively. Seed treatment tubes were placed in a rotating box and set to turn slowly overnight (18 h). EMS decontamination of the seed was achieved by performing 10 washes of 150 ml 0.02 % Tween 20 for 20 min per wash, turning slowly. After the final wash, seeds were transferred to KWS UK for sowing at a density of 500 seeds per 348 mm × 220 mm tray. Trays were kept at 4 °C for 2 days to stratify before transferring to glasshouse at 18 °C with 16 h light. Emergence was scored 7 days after sowing. 10,080 lines were grown on for seed from treatments predicted to contain a good mutation load but still maintain viability: 4,578 0.4 % EMS lines, 4,410 0.6 % EMS lines and 1,092 0.8 % EMS lines.

### M_2_ population growth

Two 17-cm spaced double rows of seed for each M_2_ line were drilled for 7,684 lines (2,738 0.4 % EMS, 3,873 0.6 % EMS and 1,073 0.8 % EMS) distributed between two sites within the UK (Newmarket and Cambridge) and a site in France (Boissay). One plant per double row was labelled, bagged for seed production and leaf material was collected. DNA isolation was carried out using the DNeasy Plant 96 Qiagen Kit for 96 samples following the manufacturer’s instructions (Qiagen, UK). M_3_ seed was collected, threshed and deposited within the John Innes Centre seed-store (1.5 °C, 7–10 % relative humidity) to ensure their long-term viability.

### Screening of the M_2_ population for *BnaC.FAD2a* mutations

A 1,212-bp *BnaC.FAD2.b* specific amplicon was amplified from 3000 seeds of the 0.8 and 0.6 % treated EMS line using the primers GTCTCCTCCCTCCAAAAAGT and CAAGACGACCAGAGACAGC with the standard PCR recipe detailed for homologue sequencing on the following cycle: 94 °C for 5 min, 35 cycles of 94 °C for 30 s (ramp 0.5 °C/s), 57 °C for 30 s (ramp 0.5 °C/s) and 72 °C for 1 min (ramp 0.5 °C/s), then 72 °C for 10 min, and store at 8 °C. Unincorporated primer and dNTPs were removed from 10 μl of the product by SAPEXO treatment [1 μl shrimp alkaline phosphotase (Roche Cat. No. 04898133001) and 0.5 μl exonuclease 1 (EXO) (GE Healthcare Cat. No. E700732)], samples incubated at 37 °C for 30 min, and denatured at 80 °C for 10 min before sequencing was performed on 1 μl of product using the BigDye Terminator v3.1 cycle sequencing kit (Applied Biosystems Catalogue number 100 reactions 4337455) according to the manufacturer’s instructions. Mutations were scored by electropherogram alignment to wild-type Cabriolet using MutationSurveyor v2.61 software (SoftGenetics, State College, PA, USA) (Dong and Yu 2011).

### Growth and phenotyping of M_3_ lines

12 M_3_ seeds were sown for each line at KWS and DNA prepared from a young leaf for each line as for the M_2_. Sequencing of *BnaC.FAD2.a* PCR product as detailed above was performed to select homozygous and wild-type out-segregant lines for phenotyping. Multiple homozygotes of each line and outsegregant examples, where available, were grown in long-day glasshouse conditions (16-h photoperiod and 18 °C/14 °C day/night) in a randomised split block design. A maximum of twelve replicate plants of three lines, Tapidor, Cabriolet and V141, were used as standard controls. Phenotyping for fatty acid profile was performed by gas chromatography (conducted at KWS) using standard industry protocols.

## Results

### Characterisation of the *BnaFAD2* family in Tapidor and Cabriolet

BAC clones derived from genomic DNA of *B. napus* var. Tapidor that had been identified previously by Smooker et al. (2011) as containing sequences that hybridised to *FAD2*-specific probes were sequenced. The results confirmed the presence of four orthologues in *B. napus*, as suggested by Yang et al. (2012b). Three copies had been positioned previously by linkage mapping (*BnaA.FAD2.a* on linkage group A5, *BnaC.FAD2.b* on C1 and *BnaC.FAD2.a* on C5; Smooker et al. 2011). We positioned the fourth (*BnaA.FAD2.b*) on linkage group A1 based on sequence similarity with the *B. rapa* genome sequence (Wang et al. 2011). These positions corresponded to those previously indicated, based on restriction fragment length polymorphism mapping, by Scheffler et al. (1997). Alignment of the coding regions of the genes showed a high level of sequence conservation between the pairs of homoeologous genes, i.e. those on A1 and C1 (95 %), and those on A5 and C5 (96 %) (see Supplemental Figure 1). The Tapidor allele of *BnaA.FAD2.b*, as with the allele in the Chinese germplasm characterised by Yang et al. (2012b), contained deletion and insertion events predicted to result in frame shifts and a truncated protein. The other three copies are predicted to encoded full-length proteins of 383 amino acids (see Supplemental Figure 2). All copies were found to be expressed in the seed, using both RT-PCR and mRNAseq (data not shown).

Oil from the rapeseed variety Cabriolet contains ~75 % oleic and 16 % PUFAs, compared with ~60 % oleic and 29 % PUFAs in variety Tapidor, suggesting that fewer *FAD2* orthologues may be functional in Cabriolet than in Tapidor. We used locus-specific PCR amplification and sequencing to characterise the family of *FAD2* orthologues in Cabriolet, with primer design based on the sequences of the Tapidor alleles. Sequence analysis revealed the same frame shifts to be present in the Cabriolet allele of *BnaA.FAD2.b*. A 1-bp deletion was identified in the Cabriolet allele of *BnaA.FAD2.a*, predicted to result in a frame shift and a truncated protein. We were unable to amplify *BnaC.FAD2.b* from Cabriolet and could detect no expression by mRNAseq analysis of RNA from seeds 45 DAP, suggesting deletion of this gene. Thus only *BnaC.FAD2.a* appears to encode a functional protein in Cabriolet.

### Development of an EMS-induced mutation population

As we wished to test the hypothesis that *BnaC.FAD2.a* is the only functional copy of the gene family in Cabriolet, we induced mutations in this genetic background. Approximately 33,000 seeds of variety Cabriolet were treated with a range of doses of EMS and from them a population, which we called the JBnaCAB_E population, was developed. Observation of seedling emergence from the treated seeds showed that treatment with increasing EMS concentration generally resulted in reduced seeding emergence and vigour, with 2 % EMS resulting in no viable seedlings. Treatments with 0.4, 0.6 and 0.8 % EMS were chosen to be grown on to produce the population. Emergence, fertility and seed number details are shown in Supplemental Table 3. Approximately 20 seeds were collected from each of these M_1_ generation plants.

The M_2_ generation was grown, with subsets of the population grown each at one of three sites (Thriplow and Cowlinge in UK and Boissay in France). All of the ~20 seeds from each M_1_ plant were sown, together, but only one plant was bagged for seed collection. Duplicate leaf tissue samples were taken from the bagged plants and DNA was prepared from one sample. The resulting population (as packets of M_3_ generation seeds derived from individual M_2_ generation plants) comprised 1,678, 3,441 and 1,037 lines resulting from treatments with 0.4, 0.6 and 0.8 % EMS, respectively. Few phenotypic abnormalities were observed within the population and no visible differences were detected between different treatment levels.

### Induced mutation of *BnaC.FAD2.a*

To search for mutations in *BnaC.FAD2.a*, we designed a 1,212-bp locus-specific PCR amplicon. After checking specificity, we first assessed the rates of mutations induced by the various EMS treatments by amplification using the DNA prepared from leaf samples as template. To do this, we amplified the 1,212-bp region of *BnaC.FAD2.a* and sequenced the PCR products (capillary sequencing). This revealed mutation rates of 1.3 % (4/302), 2.6 % (7/274) and 4.9 % (14/288) for 0.4, 0.6 and 0.8 % EMS treatments, respectively, showing the expected increasing mutation load with increasing severity of EMS treatment. To complete the screening of the population, further lines (drawn from 0.6 to 0.8 % EMS treatment) were screened by amplification and sequencing of the 1,212-bp region of *BnaC.FAD2.a*. In all, a subset of ~3,000 lines was screened. Where mutations were identified in a line, 12 seeds from the line (M_3_ generation) were sown and tested for the presence of the detected mutation by PCR amplification and sequencing using DNA purified from leaf samples taken from individual seedlings. The sequence trace files were analysed, enabling robust assessment of whether mutations were present in homozygous state (the trace file shows a clean call of the altered base) or heterozygous state (approximately half-height peaks are present in the traces at the position of the altered base). In total, 102 mutations were identified and confirmed in *BnaC.FAD2.a*. Of these, five are predicted to result in the introduction of premature stop codons, 52 are predicted to result in amino acid substitutions and 45 are predicted to be silent. All mutations detected are shown in Supplemental Table 4.

### Phenotypic analysis of lines with mutations induced in *BnaC.FAD2.a*

The subset of lines containing non-silent homozygous *BnaC.FAD2.a* mutations was assessed for effects on oil composition. To do this, the M_3_ plants used for confirmation of mutations were grown on in an unbalanced randomised block design under glasshouse conditions. The number of homozygous mutant individuals available varied between lines, from one to six (depending upon segregation in the M_2_ generation; hence the necessity of an unbalanced design). Most lines were grown on to maturity, including heterozygous and a proportion of outsegregant wild types for lines with confirmed mutations. Control lines included Tapidor, as a representative of conventional rapeseed (~60 % C18:1, 29 % PUFAs), Cabriolet as the background for mutation (~75 % C18:1, 16 % PUFAs) and V141, as a representative of the state-of-the-art high oleic lines produced by commercial breeding (~79 % C18:1, 10 % PUFAs).

**Fig. 1:**
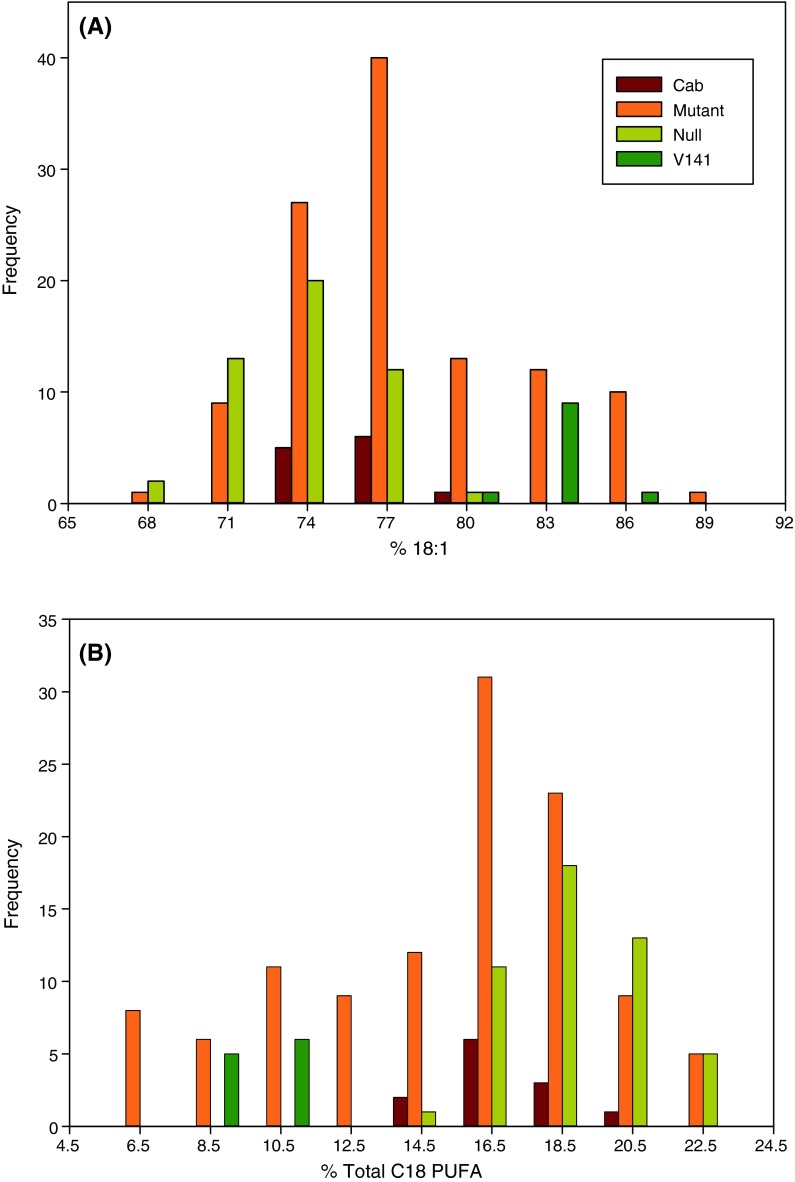
Distribution of oleic acid and 18-carbon polyunsaturated fatty acid composition of seeds in mutant and control lines. **a** Oleic acid (C18:1), **b** total 18-carbon polyunsaturated fatty acid (*Total C18 PUFA*) in seeds of *B. napus* variety Cabriolet (*Cab*), *BnaA.FAD2.a* mutated lines (*Mutant*), outsegregant lines (*Null*) and a commercial high oleic, low linolenic line (*V141*)

Analysis of the Cabriolet controls showed no line-by-block interactions and no significant differences for content of any fatty acid. Analysis of outsegregant lines not inheriting *BnaC.FAD2.b* mutations showed no line-by-block interactions but did show modest, but significant (*P* < 0.05 unless stated), differences for C16:0, C16:1 (*P* < 0.01), C18:1, C18:3, C20:2 (*P* < 0.01) and C24:1. The greater variability of outsegregants than the original genotype is likely a consequence of the very large number of background mutations segregating in lines. Similar variability would likely be superimposed on effects arising from mutation of *BnaC.FAD2.a*. As shown in Fig. 1 (for C18:1 and PUFA content) and Supplemental Figure 3 (for all other major fatty acids measured), the range of phenotypes for individual plants homozygous for mutation of *BnaC.FAD2.a* exceeds that for outsegregants, with notable skew towards higher C18:1 and lower PUFA content.

**Fig. 2:**
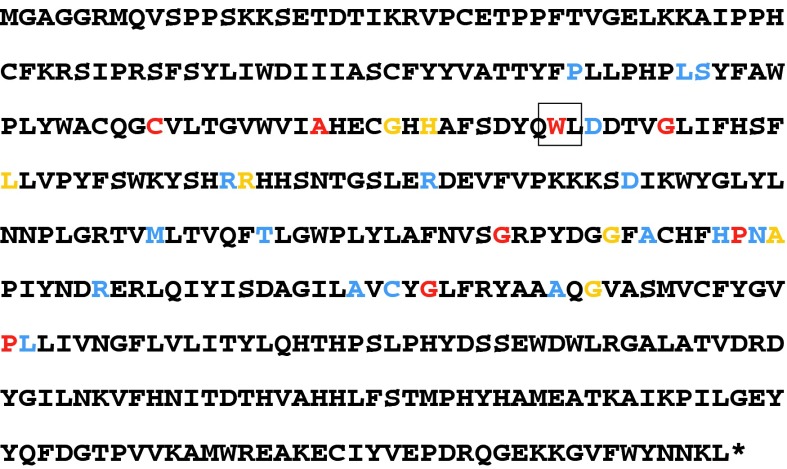
Phenotypic effects of altering amino acids in the desaturase encoded at *BnaC.FAD2.a*. *Red* mutation results in polyunsaturated fatty acid content below 7 % and oleic acid content over 80 %. *Blue* mutation results in fatty acid composition similar to wild type. *Orange* mutation results in intermediate fatty acid composition. The amino acid position mutated to a stop codon in line M0643 is *boxed*

A breakdown of mean composition for the fatty acids quantified is shown in Table 1 for 36 lines showing non-silent mutations of *BnaC.FAD2.a*, along with those for Tapidor, Cabriolet and V141. Many mutations result in little or no difference in the content of C18:1 or PUFAs. However, the mutation predicted to result in a stop codon and premature termination of the protein encoding the desaturase enzyme, along with seven further mutations, resulted in marked decreases in PUFAs, to ~6 % (compared with ~16 % for Cabriolet), and marked increase in C18:1, to ~84 % (compared with ~75 % for Cabriolet). We can exclude the possibility of the observed phenotype being caused by mutation of a gene other than *BnaC.FAD2.a* (i.e. a second site mutation) as it occurs in many independently-derived lines, even any two of which are highly unlikely to share mutations of another locus. The results of the analysis of the oil composition of lines heterozygous for these eight mutations are shown in Supplemental Figure 4. Eight other mutations resulted in intermediate phenotypes (ranges 8.5–12 % PUFAs; 79–82 % C18:1). The protein sequence of *BnaC.FAD2.a* showing the positions of all mutations is shown in Fig. 2. The mutations induced in *BnaC.FAD2.a*, as expected, had no impact on either content of saturated fatty acids (C14:0, C16:0, C18:0) or very long chain fatty acids (C20–24). In contrast to the fatty acid composition of seeds of V141, which contain around twice as much C18:2 as C18:3 (presumably caused by mutation of another locus, perhaps an orthologue of *FAD3*), the reduction in total PUFA content in mutations of *BnaC.FAD2.a* is accompanied by an increase in the proportion of C18:3 to C18:2, resulting in around two-thirds the amount of C18:2 as C18:3 in *BnaC.FAD2.a* mutant lines with PUFA content similar to, or lower than, that of V141.

**Table 1:** Oil profiles of homozygous *BnaC.FAD2.a* mutants and controls

Line	Mutation	Position relative to first coding base	Amino acid number	Original amino acid	New amino acid	No. of replicates	Mean fatty acid content (%)							
							C14:0	C16:0	C16:1	C18:0	C18:1	C18:2	C18:3	C20:0
M0830	C>T	776	259	Pro	Leu	1	0.1	3.7	0.4	1.4	86.4	1.8	2.8	0.5
M0814	G>A	310	104	Ala	Thr	2	0.0	3.8	0.3	1.3	85.4	2.2	3.5	0.6
K0472	G>A	284	95	Cys	Tyr	2	0.1	4.1	0.5	1.5	84.4	2.3	3.6	0.6
S0092	G>A	598	200	Gly	Arg	1	0.1	4.2	0.5	1.1	84.2	2.4	3.7	0.5
K0692	G>A	367	123	Gly	Ser	1	0.1	3.8	0.5	1.3	84.0	2.5	4.2	0.6
M2444	C>T	637	213	Pro	Ser	1	0.1	3.9	0.3	1.4	84.0	2.5	4.4	0.5
M0643	G>A	350	117	Trp	STOP	1	0.1	4.0	0.5	1.5	83.9	2.4	3.5	0.7
K0047	G>A	716	239	Gly	Asp	1	0.2	4.1	0.5	1.3	83.3	2.3	4.1	0.6
V141						10	0.1	4.0	0.4	1.4	82.3	5.8	2.8	0.5
S0619	G>A	743	248	Gly	Glu	6	0.1	3.9	0.4	1.3	82.2	3.2	5.4	0.6
M2234	G>A	322	108	Gly	Ser	1	0.1	3.8	0.4	1.6	82.1	3.2	5.3	0.6
S0418	C>T	328	110	His	Tyr	2	0.1	3.9	0.4	1.1	80.4	4.8	6.1	0.5
S0032	G>A	617	206	Gly	Asp	3	0.1	3.9	0.4	1.3	80.1	4.1	6.5	0.5
M2326	C>T	388	130	Leu	Phe	2	0.1	4.2	0.5	1.5	79.7	3.9	5.8	0.7
M2547	G>A	643	215	Ala	Thr	3	0.1	4.0	0.4	1.4	79.3	4.8	6.2	0.6
M2515	G>A	616	206	Gly	Tyr	1	0.1	4.4	0.4	1.6	79.2	5.6	6.4	0.4
M2179	G>A	428	143	Arg	His	6	0.1	4.1	0.5	1.5	79.0	4.3	6.3	0.7
M2147	C>T	778	260	Leu	Phe	3	0.1	4.4	0.5	1.2	75.6	6.7	8.0	0.5
S0265	C>A	642	214	Asn	Lys	6	0.1	4.5	0.4	1.4	75.5	6.7	8.0	0.6
S0017	G>A	355	119	Asp	Asn	2	0.1	4.9	0.6	1.1	75.4	5.9	7.8	0.6
K0325	G>A	543	181	Met	Ile	1	0.1	4.4	0.4	1.5	75.3	9.1	6.0	0.6
M2552	G>A	710	237	Cys	Tyr	2	0.1	5.0	0.4	1.8	75.2	6.4	7.3	0.7
M2381	G>A	715	239	Gly	Ser	4	0.1	4.3	0.3	1.5	75.1	8.5	6.9	0.6
M2127	C>T	623	208	Ala	Val	4	0.1	4.4	0.4	1.2	74.9	7.6	8.1	0.5
M0405	C>T	704	235	Ala	Val	6	0.1	4.4	0.4	1.4	74.7	9.4	6.4	0.5
K0430	G>A	490	164	Asp	Asn	2	0.1	4.4	0.4	1.1	74.6	8.3	8.0	0.5
CAB						12	0.1	4.4	0.3	1.4	74.6	8.8	7.2	0.6
M2248	C>T	737	246	Ala	Val	6	0.1	4.5	0.3	1.3	74.5	9.0	7.1	0.5
K0143	G>A	425	142	Arg	Gln	5	0.1	4.5	0.4	1.5	74.2	9.2	7.0	0.6
S0305	C>T	623	208	Ala	Val	2	0.1	4.3	0.5	1.3	74.1	8.5	7.8	0.5
M0529	C>T	241	81	Leu	Phe	6	0.1	4.4	0.4	1.5	74.0	8.9	7.6	0.5
M0014	C>T	560	187	Thr	Asn	1	0.1	4.5	0.4	1.3	72.8	10.1	7.6	0.5
S0350	C>T	634	212	His	Tyr	4	0.1	4.8	0.4	1.3	72.5	10.1	7.6	0.5
S0388	C>T	245	82	Ser	Phe	1	0.1	4.3	0.4	1.2	72.4	11.0	7.3	0.5
S0713	C>T	224	75	Pro	Leu	5	0.1	4.8	0.5	1.3	72.4	8.7	8.8	0.5
M2210	C>T	737	246	Ala	Val	4	0.1	4.8	0.5	1.3	72.4	10.3	7.5	0.5
S0354	G>A	662	221	Arg	His	6	0.1	4.5	0.4	1.3	70.7	10.8	9.0	0.5
M1070	G>A	458	153	Arg	Lys	2	0.1	4.8	0.4	1.2	69.4	11.7	9.3	0.5
TAP						12	0.1	5.0	0.3	1.8	60.4	21.7	7.7	0.7

Line	Mutation	Position relative to first coding base	Amino acid number	Original amino acid	New amino acid	No. of replicates	Mean fatty acid content (%)							
							C20:1	20:2	C22:0	C22:1	C24:0	C24:1	C18:2+C18:3	C18:3/C18:2
M0830	C>T	776	259	Pro	Leu	1	1.5	0.0	0.3	0.0	0.2	0.2	4.6	1.57
M0814	G>A	310	104	Ala	Thr	2	1.5	0.0	0.3	0.0	0.2	0.1	5.7	1.59
K0472	G>A	284	95	Cys	Tyr	2	1.5	0.0	0.3	0.0	0.2	0.1	5.9	1.55
S0092	G>A	598	200	Gly	Arg	1	1.6	0.0	0.4	0.0	0.2	0.1	6.1	1.52
K0692	G>A	367	123	Gly	Ser	1	1.6	0.0	0.4	0.0	0.3	0.1	6.7	1.66
M2444	C>T	637	213	Pro	Ser	1	1.4	0.1	0.3	0.1	0.2	0.1	6.9	1.79
M0643	G>A	350	117	Trp	STOP	1	1.7	0.1	0.4	0.0	0.3	0.2	6.0	1.44
K0047	G>A	716	239	Gly	Asp	1	1.7	0.0	0.4	0.1	0.3	0.2	6.4	1.83
V141						10	1.4	0.0	0.3	0.0	0.3	0.1	8.6	0.48
S0619	G>A	743	248	Gly	Glu	6	1.6	0.0	0.3	0.1	0.2	0.1	8.5	1.70
M2234	G>A	322	108	Gly	Ser	1	1.4	0.1	0.3	0.1	0.2	0.1	8.5	1.64
S0418	C>T	328	110	His	Tyr	2	1.5	0.1	0.3	0.0	0.2	0.1	10.9	1.26
S0032	G>A	617	206	Gly	Asp	3	1.6	0.1	0.3	0.0	0.2	0.2	10.6	1.58
M2326	C>T	388	130	Leu	Phe	2	1.8	0.0	0.4	0.1	0.3	0.2	9.7	1.49
M2547	G>A	643	215	Ala	Thr	3	1.6	0.1	0.4	0.0	0.2	0.2	11.0	1.36
M2515	G>A	616	206	Gly	Tyr	1	0.9	0.0	0.1	0.1	0.1	0.1	12.0	1.15
M2179	G>A	428	143	Arg	His	6	1.7	0.0	0.4	0.0	0.3	0.2	10.7	1.46
M2147	C>T	778	260	Leu	Phe	3	1.5	0.1	0.3	0.1	0.2	0.2	14.7	1.23
S0265	C>A	642	214	Asn	Lys	6	1.5	0.0	0.3	0.0	0.2	0.2	14.7	1.19
S0017	G>A	355	119	Asp	Asn	2	1.7	0.0	0.5	0.0	0.5	0.3	13.7	1.33
K0325	G>A	543	181	Met	Ile	1	1.3	0.1	0.3	0.0	0.2	0.2	15.1	0.65
M2552	G>A	710	237	Cys	Tyr	2	1.6	0.0	0.4	0.0	0.3	0.2	13.7	1.14
M2381	G>A	715	239	Gly	Ser	4	1.4	0.1	0.3	0.0	0.2	0.1	15.4	0.81
M2127	C>T	623	208	Ala	Val	4	1.4	0.1	0.3	0.0	0.2	0.2	15.7	1.06
M0405	C>T	704	235	Ala	Val	6	1.3	0.1	0.3	0.0	0.2	0.1	15.8	0.69
K0430	G>A	490	164	Asp	Asn	2	1.4	0.1	0.3	0.0	0.2	0.1	16.3	0.97
CAB						12	1.4	0.1	0.3	0.0	0.2	0.1	16.0	0.81
M2248	C>T	737	246	Ala	Val	6	1.3	0.1	0.3	0.0	0.2	0.1	16.1	0.79
K0143	G>A	425	142	Arg	Gln	5	1.3	0.1	0.3	0.0	0.2	0.2	16.2	0.76
S0305	C>T	623	208	Ala	Val	2	1.4	0.1	0.3	0.1	0.2	0.2	16.3	0.92
M0529	C>T	241	81	Leu	Phe	6	1.3	0.1	0.3	0.0	0.2	0.1	16.5	0.85
M0014	C>T	560	187	Thr	Asn	1	1.3	0.1	0.3	0.0	0.2	0.1	17.6	0.75
S0350	C>T	634	212	His	Tyr	4	1.3	0.1	0.3	0.0	0.2	0.1	17.7	0.76
S0388	C>T	245	82	Ser	Phe	1	1.5	0.1	0.3	0.0	0.2	0.2	18.3	0.66
S0713	C>T	224	75	Pro	Leu	5	1.4	0.1	0.3	0.0	0.2	0.2	17.6	1.02
M2210	C>T	737	246	Ala	Val	4	1.4	0.1	0.3	0.0	0.2	0.2	17.8	0.73
S0354	G>A	662	221	Arg	His	6	1.3	0.1	0.3	0.0	0.2	0.2	19.8	0.84
M1070	G>A	458	153	Arg	Lys	2	1.3	0.1	0.3	0.1	0.2	0.2	21.1	0.80
TAP						12	1.1	0.1	0.3	0.0	0.2	0.1	29.4	0.35

Fatty acid composition for: myristic acid (C14:0), palmitic acid (C16:0), 11-hexadecenoic acid (C16:1), stearic acid (C18:0), oleic acid (C18:1), linoleic acid (C18:2), linolenic acid (C18:3), arachidic acid (C20:0), 9-eicosenoic acid (C20:1), eicosadienoic acid (C20:2), behenic acid (22:0), erucic acid (C22:1), cerebronic acid (C24:0) and nervonic acid (C24:1). Cultivar controls: Cabriolet wild-type (*CAB*), Tapidor (*TAP*) and V141

## Discussion

### Development of the JBnaCAB_E population

We developed an EMS mutagenised population of *B. napus* consisting of ~ 5500 lines comprising sub-populations with three different severities of EMS treatment. Despite the high mutation loads, estimated as 1.3, 2.6 and 4.9 % for 0.4, 0.6 and 0.8 % EMS treatments, respectively, we observed remarkably few visible phenotypes. In contrast, Stephenson et al. (2010) saw a wide range of visible phenotypes in a *B. rapa* TILLING population treated with 0.3 % EMS and estimated to carry a 2.7 % mutation load. The difference is likely due to the much greater genetic redundancy in *B. napus*, which is a recently-formed allotetraploid in which one genome was inherited from a *B. rapa* progenitor and the other from a *B. oleracea* progenitor. This observation does not mean that there is no upper limit to the EMS treatment that *B. napus* can withstand. For example, treatment with 2 % EMS resulted in failure of seeds to germinate and establish. In addition, the higher level of background mutations in more severely mutated material may require more extensive backcrossing to re-establish vigour. Nevertheless, the JBnaCAB_E population represents an excellent resource for the genetic analysis of traits in a winter oilseed rape crop type of *B. napus*.

### Functional characterisation of the *BnaFAD2* family

Our analysis of the *BnaFAD2* gene family in variety Cabriolet revealed the presence of only one functional member of the family, *BnaC.FAD2.a*. As we are aware of no reports of additivity for *FAD2* family members or co-dominance of mutant alleles, we hypothesised that the reduced PUFA phenotype in Cabriolet compared with conventional rapeseed cultivars, such as Tapidor, may be the result of loss of function at three loci revealing the reduced potency of the enzyme encoded by the one remaining locus, *BnaC.FAD2.a*. To test whether enzyme function could be modulated in this way, we produced a large allelic series of mutations of this locus by severe EMS treatment of the Cabriolet genotype. The result was, indeed, lines exhibiting a range of phenotypes, with the most severely affected having the phenotype ~84 % C18:1, ~6 % PUFAs. This phenotype matches that found to be the result of RNAi knock-down of the gene family in *B. napus* (Peng et al. 2010), with the remaining PUFAs likely originating from the plastidial biosynthesis pathway. In addition, there are several genotypes with intermediate oil compositions, consistent with further impairment (but not abolition) of function. Thus our results confirm that *B. napus* contains four orthologues of *FAD2*, that the breeders in achieving the reduced PUFA phenotype observed in Cabriolet have brought together knock-out mutations of three copies and the final functional copy is susceptible to modulation of function by EMS mutagenesis.

We have produced a series of mutations that enable assessment of the functional significance of specific amino acids in the linoleate desaturase encoded by *BnaC.FAD2.a*. Before these assessments can be performed rigorously, it will be necessary for the lines to undergo an extensive program of backcrossing to reduce background mutations that might affect the phenotype. In the meantime, however, our results provide indications of those that are likely to be critical (i.e. those for which we have detected a change in oil composition) or unlikely to be so important (i.e. those at which mutations result in little or no change in oil composition). None of the lines produced detectable quantities of unusual fatty acids.

### Novel rapeseed oils

By targeting specifically *BnaC.FAD2.a* in variety Cabriolet, we have produced an allelic series of lines with variation in the proportions of C18:1 and PUFAs in their oil. When produced on a sufficient scale, these novel types of rapeseed oil may have important uses.

The principal effect on the physical properties of reducing the PUFA content of rapeseed oil is anticipated to be an increase in its thermal stability, as saturated fatty acids (SFA) and MUFAs are more stable than PUFAs. This will make it more suitable not only for high-temperature cooking, but also for high-temperature industrial applications, such as engine lubricants and hydraulic fluids, as a substitute for mineral oil. It is noteworthy that even in the lines with the lowest PUFA content, the SFA content of the oil has not increased (which is good as SFAs tend to increase the temperature at which oils begin to set) and that C18:3 has been reduced only modestly (which is good as C18:3 is linked to cold tolerance).

Dietary studies into the benefits of PUFAs in the diet are confounded by a lack of suitable variation within a type of vegetable oil. The allelic series produced by mutation of *BnaC.FAD2.a* could be used to produce a panel of rapeseed oils with varying PUFA contents. Lines with very similar C18:1 and overall PUFA contents to V141, such as S0619, could be used in combination with oil from V141 to test the effects of varying proportions of C18:2 and C18:3 in the diet.

Although the very high C18:1 phenotype that we have produced by mutation could also be produced by RNAi, there are important advantages associated with genetic variation induced by mutation breeding over that induced by genetic modification (GM) technology. In particular, the cost of completing the regulatory process for GM crops is prohibitive. There are no such costs for material produced by mutation breeding. The RNAi approach, particularly if seed-specific targeting of the silencing was incorporated, may reduce any tendency to cold susceptibility. However, the high oleic lines we have produced grow successfully over the winter in UK conditions, so cold susceptibility appears not to be a problem.

## Conclusions

The genome redundancy arising from polyploidy makes the genetic study of traits, necessary for predictive breeding, challenging in many crop species. Our study demonstrates that quantitative reduction in PUFA content of oil in oilseed rape variety Cabriolet is the consequence of breeders selecting and combining knock-out alleles, as has been observed for other traits in rapeseed that have been the focus of breeding, such as seed glucosinolate content (Harper et al. 2012). The phenotypic values of such traits can now be extended in a predictive way in winter oilseed rape, by targeting further relevant loci using the JBnaCAB_E population. By thoroughly understanding the genetic basis of PUFA content of rapeseed oil, we successfully knocked out the remaining functional orthologue of *FAD2*, resulting in an oil composition very high in C18:1 and very low in PUFAs. By achieving it in this way, the newly-developed crop type can be cultivated for applications such as renewable alternatives to mineral oils as lubricants without the regulatory costs associated with transgenic approaches.

## Electronic supplementary material

Below is the link to the electronic supplementary material.

Supplementary material 1 (PDF 631 kb)
