# Supplementary material for: The control of seed oil polyunsaturate content in the polyploid crop species Brassica napus
Source: Mol Breed. 2013 Sep 21;33(2):349–62. doi: 10.1007/s11032-013-9954-5 (PMC3901927; doi:10.1007/s11032-013-9954-5)
Supplement: Supplementary file 1 — Supplementary material 1 (PDF 631 kb) [file 11032_2013_9954_MOESM1_ESM.pdf]

The control of seed oil polyunsaturate content in the polyploid crop species *Brassica napus*

Molecular Breeding

Rachel Wells, Martin Trick, Eleni Sompourou, Leah Clissold, Colin Morgan, Peter Werner, Carl Gibbard, Matthew Clarke, Richard Jennaway, and Ian Bancroft

Corresponding Author:

Prof. Ian Bancroft
CNAP/M1
Department of Biology,
University of York,
Wentworth Way,
Heslington,
York
YO10 5DD

Telephone: +44 (0) 1904 328778
E-Mail: ian.bancroft@york.ac.uk
Skype: prof.ian.bancroft

Primer No.	Sequence (5'-3')	Length (bp)	GC %	Tm
1	GTCTCCTCCCTCCAAAAAGT	20	50.0	54.9
2	GTGTCTCCTCCCTCCAAA	18	55.6	51.9
3	CTACAGAAACAAACATGGGC	20	45.0	53.1
4	CTCTCCTCCCTCCAGCTCCC	20	70.0	62.4
5	CTCTTCGACATCCTCCTCTC	20	55.0	53.3
6	CCTCGTCCCTTACTTCTCCTG	21	57.1	58.2
7	CCTCATAACTTATTGTTGTACCAG	24	37.5	53.9
8	CAAGACGACCAGAGACAGC	20	55.3	55.0
9	GAACTCGACAAATTTGCCTG	20	55.7	45.0

Supplemental Table 1. Primers used for the amplification of *BnaFAD2.*

Homologue	F primer	R primer	Amplicon size (bp)	Notes
*Bna*C.*FAD2*.a	1	8	1212	
BnaA.*FAD2*.a	2	7	1133	
*Bna*C.*FAD2*.b	5	7	991	Will not amplify in Cabriolet
BnaA.*FAD2*.b	6	9	966	
BnaA.*FAD2*.b	3	7	1173	*Bna*A.*FAD2*.a-specific in Cabriolet. Will also amplify *Bna*C.*FAD2*.a in other genotypes.
BnaA.*FAD2*.b	4	7	1133	*Bna*A.*FAD2*.a-specific in Cabriolet. Will also amplify *Bna*C.*FAD2*.a in other genotypes.

Supplemental Table 2. Primer combinations required for the homologue specific amplification of *BnaFAD2.*

Treatment	Total sown	Establishment %	No. grown on	% plants with >20seed	Fertile plants %
0	1000	86.0	42	73.2	97.6
0.2	6000	77.0	42	88.1	100.0
0.4	6000	76.9	4130	91.6	90.2
0.6	6000	73.5	4352	92.0	98.7
0.8	6000	60.7	1148	93.7	100.0
1	6000	70.0	42	63.3	71.4
2	1000	0.0	0	N/A	N/A
0	1000	86.0	42	73.2	97.6

Supplemental Table 3. Emergence, fertility and seed number of *B. napus* Cabriolet seeds subjected to EMS treatment.

Line		EMS %	Mutation	Status	Position relative to first coding base	Amino acid number	Amino acid	Amino acid classification	New amino acid	New amino acid classification
S	713	0.6	C>CT	HET	224	75	Pro	Non-polar	Leu	Non-polar
M	77	0.4	C>CT	HET	228	76	Leu	Silent		
S	595	0.6	C>CT	HET	228	76	Leu	Silent		
K	28	0.8	C>CT	HET	239	80	Pro	Non-polar	Leu	Non-polar
M	529	0.6	C>T	HOM	241	81	Leu	Non-polar	Phe	Non-polar
S	388	0.8	C>CT	HET	245	82	Ser	Polar	Phe	Non-polar
S	314	0.8	G>AG	HET	257	86	Trp		STOP	
M	2505	0.8	G>AG	HET	258	86	Trp		STOP	
K	722	0.6	C>CT	HET	264	88	Leu	Silent		
M	2419	0.8	C>CT	HET	264	88	Leu	Silent		
S	732	0.6	G>AG	HET	270	90	Trp		STOP	
K	472	0.8	G>GA	HET	284	95	Cys	Polar	Tyr	Polar
M	2464	0.8	G>A	HOM	286	96	Val	Non-polar	Ile	Non-polar
M	1069	0.6	G>AG	HET	296	99	Gly	Polar	Asp	Acidic
S	127	0.8	C>CT	HET	297	99	Gly	Silent		
M	814	0.6	G>A	HOM	310	104	Ala	Non-polar	Thr	Polar
K	164	0.6	C>CT	HET	312	104	Ala	Silent		
S	635	0.6	C>CT	HET	315	105	His	Silent		
S	1021	0.6	G>AG	HET	316	106	Glu	Acidic	Lys	Basic
S	135	0.8	C>CT	HET	321	107	Cys	Silent		
M	2234	0.8	G>GA	HET	322	108	Gly	Polar	Ser	Polar
S	418	0.8	C>CT	HET	328	110	His	Basic	Tyr	Polar
M	951	0.6	C>T	HOM	330	110	His	Silent		
S	422	0.8	C>CT	HET	333	111	Ala	Silent		
M	888	0.6	C>CT	HET	345	115	Tyr	Silent		
M	643	0.6	G>AG	HET	350	117	Trp		STOP	
S	17	0.8	G>AG	HET	355	119	Asp	Acidic	Asn	Polar
S	57	0.8	C>CT	HET	366	122	Val	Silent		
K	692	0.6	G>GA	HET	367	123	Gly	Polar	Ser	Polar
S	149	0.8	G>AG	HET	367	123	Gly	Polar	Ser	Polar
K	647	0.4	G>GA	HET	368	123	Gly	Polar	Asp	Polar
S	740	0.6	C>CT	HET	375	125	Ile	Silent		
M	2326	0.8	C>CT	HET	388	130	Leu	Non-polar	Phe	Non-polar
M	830	0.6	C>CT	HET	390	130	Leu	Silent		
K	71	0.8	C>CT	HET	405	135	Phe	Silent		
S	131	0.8	C>T	HOM	408	136	Ser	Silent		
S	689	0.6	C>CT	HET	408	136	Ser	Silent		
S	1015	0.6	C>CT	HET	408	136	Ser	Silent		
K	60	0.8	C>CT	HET	417	139	Tyr	Silent		
K	350	0.6	C>CT	HET	421	141	His	Basic	Tyr	Polar
K	143	0.6	G>A	HOM	425	142	Arg	Basic	Gln	Polar
M	2179	0.8	G>A	HOM	428	143	Arg	Basic	His	Basic
M	1112	0.6	C>CT	HET	432	144	His	Silent		
S	322	0.8	C>CT	HET	437	146	Ser	Polar	Phe	Non-polar
M	1083	0.6	C>CT	HET	438	146	Ser	Silent		
M	962	0.6	G>GA	HET	458	153	Arg	Basic	Lys	Basic
M	1069	0.6	G>AG	HET	458	153	Arg	Basic	Lys	Basic
M	1070	0.6	G>AG	HET	458	153	Arg	Basic	Lys	Basic
M	635	0.6	C>CT	HET	475	159	Pro	Non-polar	Ser	Polar
M	611	0.6	G>A	HOM	480	160	Lys	Silent		
K	430	0.8	G>GA	HET	490	164	Asp	Acidic	Asn	Polar
K	750	0.6	G>GA	HET	498	166	Lys	Silent		
M	2345	0.8	G>AG	HET	510	170	Lys	Silent		
K	325	0.6	G>A	HOM	543	181	Met	Non-polar	Ile	Non-polar
M	14	0.4	C>CT	HET	560	187	Thr	Polar	Asn	Polar
M	2466	0.8	G>AG	HET	566	189	Gly	Polar	Asp	Acidic
S	108	0.8	G>AG	HET	566	189	Gly	Polar	Asp	Acidic
M	362	0.6	G>GA	HET	570	190	Trp		STOP	
K	420	0.8	C>CT	HET	579	193	Tyr	Silent		
S	92	0.8	G>AG	HET	598	200	Gly	Polar	Arg	Basic
M	965	0.6	C>CT	HET	615	205	Gly	Silent		
M	2515	0.8	G>AG	HET	616	206	Gly	Polar	Tyr	Polar
S	32	0.8	G>AG	HET	617	206	Gly	Polar	Asp	Acidic
M	2127	0.8	C>CT	HET	623	208	Ala	Non-polar	Val	Non-polar
S	305	0.8	C>CT	HET	623	208	Ala	Non-polar	Val	Non-polar
M	2287	0.8	C>CT	HET	627	209	Cys	Silent		
M	967	0.6	C>CT	HET	633	211	Phe	Silent		

M	1136	0.6	C>CT	HET	633	211	Phe	Silent		
S	350	0.8	C>CT	HET	634	212	His	Basic	Tyr	Polar
M	2444	0.8	C>CT	HET	637	213	Pro	Non-polar	Ser	Polar
K	497	0.4	C>CT	HET	642	214	Asn	Silent		
S	265	0.8	C>A	HOM	642	214	Asn	Polar	Lys	Basic
M	2547	0.8	G>AG	HET	643	215	Ala	Non-polar	Thr	Polar
S	770	0.6	C>CT	HET	657	219	Asn	Silent		
S	1154	0.6	C>CT	HET	657	219	Asn	Silent		
S	354	0.8	G>A	HOM	662	221	Arg	Basic	His	Basic
M	2481	0.8	C>CT	HET	684	228	Ile	Silent		
S	405	0.8	C>CT	HET	686	229	Ser	Polar	Phe	Non-polar
K	938	0.6	C>CT	HET	687	229	Ser	Silent		
S	585	0.6	C>T	HOM	687	229	Ser	Silent		
S	336	0.8	C>CT	HET	702	234	Leu	Silent		
M	405	0.6	C>T	HOM	704	235	Ala	Non-polar	Val	Non-polar
M	2552	0.8	G>AG	HET	710	237	Cys	Polar	Tyr	Polar
M	2381	0.8	G>AG	HET	715	239	Gly	Polar	Ser	Polar
K	47	0.8	G>AG	HET	716	239	Gly	Polar	Asp	Acidic
S	389	0.8	C>CT	HET	729	243	Tyr	Silent		
K	411	0.8	C>CT	HET	732	244	Ala	Silent		
M	2210	0.8	C>CT	HET	737	246	Ala	Non-polar	Val	Non-polar
M	2248	0.8	C>TC	HOM	737	246	Ala	Non-polar	Val	Non-polar
S	619	0.6	G>A	HOM	743	248	Gly	Polar	Glu	Acidic
M	2349	0.8	G>A	HOM	772	258	Val	Non-polar	Ile	Non-polar
M	830	0.6	C>CT	HET	776	259	Pro	Non-polar	Leu	Non-polar
S	244	0.8	C>CT	HET	776	259	Pro	Non-polar	Leu	Non-polar
S	243	0.8	G>AG	HET	777	259	Pro	Silent		
M	2147	0.8	C>CT	HET	778	260	Leu	Non-polar	Phe	Non-polar
M	2135	0.8	G>GA	HET	807	269	Leu	Silent		
M	2170	0.8	G>GA	HET	807	269	Leu	Silent		
S	1154	0.6	C>CT	HET	816	272	Tyr	Silent		
M	1085	0.6	G>AG	HET	876	292	Arg	Silent		
S	329	0.8	C>CT	HET	890	297	Thr	Polar	Ile	Non-polar
M	1020	0.6	C>CT	HET	924	308	Val	Silent		
M	2230	0.8	C>CT	HET	939	313	Thr	Silent		

Supplemental Table 4. Mutations in *BnaC.FAD2.a* identified in the JBnaCAB_E population.

Supplemental Figure 1. BnaFAD2 sequence alignment

```
                                                                      Section 1
                 (1)  1        10        20        30        40            55
Tap_BnaC.FAD2.a  (1)  ATGGGTGCAGGTGGAAGAATGCAAGTGTCTCCTCCCTCCAAGAAGTCTGAAACCG
Cab_BnaC.FAD2.a  (1)  ATGGGTGCAGGTGGAAGAATGCAAGTGTCTCCTCCCTCCAAGAAGTCTGAAACCG
Tap_BnaA.FAD2.a  (1)  ATGGGTGCAGGTGGAAGAATGCAAGTGTCTCCTCCCTCCAAAAAGTCTGAAACCG
Cab_BnaA.FAD2.a  (1)  ATGGGTGCAGGTGGAAGAATGCAAGTGTCTCCTCCCTCCAAAAAGTCTGAAACCG
Tap_BnaC.FAD2.b  (1)  ATGGGCGCAGGTGGAAGAATGCAAGTCTCTCCTCCCTCCAGCTCCCCCGAAACCA
Tap_BnaA.FAD2.b  (1)  ATGGGCGCAGGTGGAAGAATGCAAGTCTCTCCTCCCTCCAGCTCCCCCGGAACCA
Cab_BnaA.FAD2.b  (1)  ATGGGCGCAGGTGGAAGAATGCAAGTCTCTCCTCCCTCCAGCTCCCCCGGAACCA
                                                                      Section 2
                (56)  56            70        80        90        100      110
Tap_BnaC.FAD2.a (56)  ACACCATCAAGCGCGTACCCTGCGAGACACCGCCCTTCACTGTCGGAGAACTCAA
Cab_BnaC.FAD2.a (56)  ACACCATCAAGCGCGTACCCTGCGAGACACCGCCCTTCACTGTCGGAGAACTCAA
Tap_BnaA.FAD2.a (56)  ACAACATCAAGCGCGTACCCTGCGAGACACCGCCCTTCACTGTCGGAGAACTCAA
Cab_BnaA.FAD2.a (56)  ACAACATCAAGCGCGTACCCTGCGAGACACCGCCCTTCACTGTCGGAGAACTCAA
Tap_BnaC.FAD2.b (56)  AAACCCTCAAACGCGTCCCCTGCGAGACACCACCCTTCACTCTCGGAGACCTCAA
Tap_BnaA.FAD2.b (56)  ACACCCTCAAACGCGTCCCCTGCGAGACACCACCATTCACTCTCGGAGACCTCAA
Cab_BnaA.FAD2.b (56)  ACACCCTCAAACGCGTCCCCTGCGAGACACCACCATTCACTCTCGGAGACCTCAA
                                                                      Section 3
               (111)  111      120       130       140       150           165
Tap_BnaC.FAD2.a (111) GAAAGCAATCCCACCGCACTGTTTCAAACGCTCGATCCCTCGCTCTTTCTCCTAC
Cab_BnaC.FAD2.a (111) GAAAGCAATCCCACCGCACTGTTTCAAACGCTCGATCCCTCGCTCTTTCTCCTAC
Tap_BnaA.FAD2.a (111) GAAAGCAATCCCACCGCACTGTTTCAAACGCTCGATCCCTCGCTCTTTCTCCTAC
Cab_BnaA.FAD2.a (111) GAAAGCAATCCCACCGCACTGTTTCAAACGCTCGATCCCTCGCTCTTTCTCCTAC
Tap_BnaC.FAD2.b (111) GAAAGCAATCCCACCTCACTGCTTCAAACGCTCCATCCCTCGCTCCTTCTCCTAC
Tap_BnaA.FAD2.b (111) GAAAGCAATCCCACCTCACTGCTTCAAACGCTCCATCCCACGCTCCTTCTCCT-C
Cab_BnaA.FAD2.b (111) GAAAGCAATCCCACCTCACTGCTTCAAACGCTCCATCCCACGCTCCTTCTCCT-C
                                                                      Section 4
               (166)  166           180       190       200       210      220
Tap_BnaC.FAD2.a (166) CTCATCTGGGACATC--ATCATAGCCTCCTGCTTCTACTACGTCGCCACCACTTA
Cab_BnaC.FAD2.a (166) CTCATCTGGGACATC--ATCATAGCCTCCTGCTTCTACTACGTCGCCACCACTTA
Tap_BnaA.FAD2.a (166) CTCATCTGGGACATC--ATCATAGCCTCCTGCTTCTACTACGTCGCCACCACTTA
Cab_BnaA.FAD2.a (166) CTCATCTGGGACATC--ATCATAGCCTCCTGCTTCTACTACGTCGCCACCA-TTA
Tap_BnaC.FAD2.b (166) CTCCTCTTCGACATC--CTCGTCTCCTCCTCCCTCTACCACCTCTCCACAGCCTA
Tap_BnaA.FAD2.b (165) TTCGACATCATCATCTCCTCCTCGGCTCCTCCCTCTACCACCTCTCCACAGCCTA
Cab_BnaA.FAD2.b (165) TTCGACATCATCATCTCCTCCTCGGCTCCTCCCTCTACCACCTCTCCACAGCCTA
                                                                      Section 5
               (221)  221      230       240       250       260           275
Tap_BnaC.FAD2.a (219) CTTCCCTCTCCTCCCTCACCCTCTCTCCTACTTCGCCTGGCCTCTCTACTGGGCC
Cab_BnaC.FAD2.a (219) CTTCCCTCTCCTCCCTCACCCTCTCTCCTACTTCGCCTGGCCTCTCTACTGGGCC
Tap_BnaA.FAD2.a (219) CTTCCCTCTCCTCCCTCACCCTCTCTCCTACTTCGCCTGGCCTCTCTACTGGGCC
Cab_BnaA.FAD2.a (218) CTTCCCTCTCCTCCCTCACCCTCTCTCCTACTTCGCCTGGCCTCTCTACTGGGCC
Tap_BnaC.FAD2.b (219) CTTCCCTCTCCTCCCCCACCCTCTCCCTTACCTCGCCTGGCCCCTCTACTGGGCC
Tap_BnaA.FAD2.b (220) CTTCCCTCTCC---------------CTTACCTCGCCTGACCCCTCTACTGGGCC
Cab_BnaA.FAD2.b (220) CTTCCCTCTCC---------------CTTACCTCGCCTGACCCCTCTACTGGGCC
```

Supplemental Figure 1. BnaFAD2 sequence alignment

```
                                                                  Section 6
                  (276) 276          290        300        310        320       330
Tap_BnaC.FAD2.a   (274) TGCCAAGGGTGCGTCCTAACCGGCGTCTGGGTCATAGCCCACGAGTGCGGCCACC
Cab_BnaC.FAD2.a   (274) TGCCAAGGGTGCGTCCTAACCGGCGTCTGGGTCATAGCCCACGAGTGCGGCCACC
Tap_BnaA.FAD2.a   (274) TGCCAGGGCTGCGTCCTAACCGGCGTCTGGGTCATAGCCCACGAGTGCGGCCACC
Cab_BnaA.FAD2.a   (273) TGCCAGGGCTGCGTCCTAACCGGCGTCTGGGTCATAGCCCACGAGTGCGGCCACC
Tap_BnaC.FAD2.b   (274) TGCCAAGGCTGCGTCCTAACGGGCCTCTGGGTCATCGCCCACGAATGCGGCCACC
Tap_BnaA.FAD2.b   (260) TGCCAAGGCTGCGTCCTAACGGGCCTCTGGGTCATAGCCCACGAGTGCGGCCACC
Cab_BnaA.FAD2.b   (260) TGCCAAGGCTGCGTCCTAACGGGCCTCTGGGTCATAGCCCACGAGTGCGGCCACC
```

```
                                                                  Section 7
                  (331) 331      340        350        360        370            385
Tap_BnaC.FAD2.a   (329) ACGCCTTCAGCGACTACCAGTGGCTTGACGACACCGTCGGTCTCATCTTCCACTC
Cab_BnaC.FAD2.a   (329) ACGCCTTCAGCGACTACCAGTGGCTTGACGACACCGTCGGTCTCATCTTCCACTC
Tap_BnaA.FAD2.a   (329) ACGCCTTCAGCGACTACCAGTGGCTGGACGACACCGTCGGCCTCATCTTCCACTC
Cab_BnaA.FAD2.a   (328) ACGCCTTCAGCGACTACCAGTGGCTGGACGACACCGTCGGCCTCATCTTCCACTC
Tap_BnaC.FAD2.b   (329) ACGCCTTCAGCGACCACCAGTGGCTGGACGACGCCGTGGGCCTCGTCTTCCACTC
Tap_BnaA.FAD2.b   (315) ACGCCTTCAGCGACCACCAGTGGCTGGACGACGCCGTCGGCCTCGTCTTCCACTC
Cab_BnaA.FAD2.b   (315) ACGCCTTCAGCGACCACCAGTGGCTGGACGACGCCGTCGGCCTCGTCTTCCACTC
```

```
                                                                  Section 8
                  (386) 386          400        410        420        430       440
Tap_BnaC.FAD2.a   (384) CTTCCTCCTCGTCCCTTACTTCTCCTGGAAGTACAGTCATCGACGCCACCATTCC
Cab_BnaC.FAD2.a   (384) CTTCCTCCTCGTCCCTTACTTCTCCTGGAAGTACAGTCATCGACGCCACCATTCC
Tap_BnaA.FAD2.a   (384) CTTCCTCCTCGTCCCTTACTTCTCCTGGAAGTACAGTCATCGACGCCACCATTCC
Cab_BnaA.FAD2.a   (383) CTTCCTCCTCGTCCCTTACTTCTCCTGGAAGTACAGTCATCGACGCCACCATTCC
Tap_BnaC.FAD2.b   (384) CTTCCTCCTCGTCCCTTACTTCTCCTGGAAGTACAGCCATCGACGCCACCATTCC
Tap_BnaA.FAD2.b   (370) CTTCCTCCTCGTCCCGTACTTCTCCTGGAAGTACATCCAT-GACGCCACCATTCC
Cab_BnaA.FAD2.b   (370) CTTCCTCCTCGTCCCGTACTTCTCCTGGAAGTACATCCAT-GACGCCACCATTCC
```

```
                                                                  Section 9
                  (441) 441      450        460        470        480            495
Tap_BnaC.FAD2.a   (439) AACACTGGCTCCCTCGAGAGAGACGAAGTGTTTGTCCCCAAGAAGAAGTCAGACA
Cab_BnaC.FAD2.a   (439) AACACTGGCTCCCTCGAGAGAGACGAAGTGTTTGTCCCCAAGAAGAAGTCAGACA
Tap_BnaA.FAD2.a   (439) AACACTGGCTCCCTCGAGAGAGACGAAGTGTTTGTCCCCAAGAAGAAGTCAGACA
Cab_BnaA.FAD2.a   (438) AACACTGGCTCCCTCGAGAGAGACGAAGTGTTTGTCCCCAAGAAGAAGTCAGACA
Tap_BnaC.FAD2.b   (439) AACACCGGATCCCTCGAGAGGGATGAAGTGTTCGTCCCCAAGAAGAAATCCGACA
Tap_BnaA.FAD2.b   (424) AACACCGGATCCCTCGATAGGGACGAAGTGTTCGTCCCCAAGAAGAAATCCGACA
Cab_BnaA.FAD2.b   (424) AACACCGGATCCCTCGATAGGGACGAAGTGTTCGTCCCCAAGAAGAAATCCGACA
```

```
                                                                  Section 10
                  (496) 496          510        520        530        540       550
Tap_BnaC.FAD2.a   (494) TCAAGTGGTACGGCAAGTACCTCAACAACCCTTTGGGACGCACCGTGATGTTAAC
Cab_BnaC.FAD2.a   (494) TCAAGTGGTACGGCAAGTACCTCAACAACCCTTTGGGACGCACCGTGATGTTAAC
Tap_BnaA.FAD2.a   (494) TCAAGTGGTACGGCAAGTACCTCAACAACCCTTTGGGACGCACCGTGATGTTAAC
Cab_BnaA.FAD2.a   (493) TCAAGTGGTACGGCAAGTACCTCAACAACCCTTTGGGACGCACCGTGATGTTAAC
Tap_BnaC.FAD2.b   (494) TCAAGTGGTACGGAAAGTACCTCAACAACCCGCTAGGACGCACGGTGATGCTAAC
Tap_BnaA.FAD2.b   (479) TCAAGTGGTACGGCAAGTACCTCAACAACCCGCTAGGACGCACGGTGATGCTAAC
Cab_BnaA.FAD2.b   (479) TCAAGTGGTACGGCAAGTACCTCAACAACCCGCTAGGACGCACGGTGATGCTAAC
```

Supplemental Figure 1. BnaFAD2 sequence alignment

Section 11

```
                 (551) 551      560       570       580       590           605
Tap_BnaC.FAD2.a  (549) GGTTCAGTTCACTCTCGGCTGGCCGTTGTACTTAGCCTTCAACGTCTCGGGAAGA
Cab_BnaC.FAD2.a  (549) GGTTCAGTTCACTCTCGGCTGGCCGTTGTACTTAGCCTTCAACGTCTCGGGAAGA
Tap_BnaA.FAD2.a  (549) GGTTCAGTTCACTCTCGGCTGGCCTTTGTACTTAGCCTTCAACGTCTCGGGGAGA
Cab_BnaA.FAD2.a  (548) GGTTCAGTTCACTCTCGGCTGGCCTTTGTACTTAGCCTTCAACGTCTCGGGGAGA
Tap_BnaC.FAD2.b  (549) CGTCCAGTTCACGCTCGGCTGGCCGTTGTACTTAGCCTTCAACGTCTCTGGAAGA
Tap_BnaA.FAD2.b  (534) CGTCCAGTTCAAGCTCGGCTGGCCGTTGTACTTAGCCTTCAACGTCTCGGGAAGA
Cab_BnaA.FAD2.b  (534) CGTCCAGTTCAAGCTCGGCTGGCCGTTGTACTTAGCCTTCAACGTCTCGGGAAGA
```

Section 12

```
                 (606) 606           620       630       640       650      660
Tap_BnaC.FAD2.a  (604) CCTTACGACGGCGGCTTCGCTTGCCATTTCCACCCCAACGCTCCCATCTACAACG
Cab_BnaC.FAD2.a  (604) CCTTACGACGGCGGCTTCGCTTGCCATTTCCACCCCAACGCTCCCATCTACAACG
Tap_BnaA.FAD2.a  (604) CCTTACGACGGCGGCTTCGCTTGCCATTTCCACCCCAACGCTCCCATCTACAACG
Cab_BnaA.FAD2.a  (603) CCTTACGACGGCGGCTTCGCTTGCCATTTCCACCCCAACGCTCCCATCTACAACG
Tap_BnaC.FAD2.b  (604) CCTTACAGCGACGGTTTCGCTTGCCATTTCCACCCGAACGCTCCCATCTACAACG
Tap_BnaA.FAD2.b  (589) CCTTACAGCGACGGTTTCGCTTGCCATTTCCACCCGAACGCTCCCATCTACAACG
Cab_BnaA.FAD2.b  (589) CCTTACAGCGACGGTTTCGCTTGCCATTTCCACCCGAACGCTCCCATCTACAACG
```

Section 13

```
                 (661) 661      670       680       690       700           715
Tap_BnaC.FAD2.a  (659) ACCGCGAGCGTCTCCAGATATACATCTCCGACGCTGGCATCCTCGCCGTCTGCTA
Cab_BnaC.FAD2.a  (659) ACCGCGAGCGTCTCCAGATATACATCTCCGACGCTGGCATCCTCGCCGTCTGCTA
Tap_BnaA.FAD2.a  (659) ACCGTGAGCGTCTCCAGATATACATCTCCGACGCTGGCATCCTCGCCGTCTGCTA
Cab_BnaA.FAD2.a  (658) ACCGTGAGCGTCTCCAGATATACATCTCCGACGCTGGCATCCTCGCCGTCTGCTA
Tap_BnaC.FAD2.b  (659) ACCGCGAGCGTCTCCAGATATACATCTCTGACGCTGGCGTCCTCTCCGTATGTTA
Tap_BnaA.FAD2.b  (644) ACCGCGAGCGTCTCCAGATATACATCTCTGACGCTGGCGTCCTCTCCGTATGTTA
Cab_BnaA.FAD2.b  (644) ACCGCGAGCGTCTCCAGATATACATCTCTGACGCTGGCGTCCTCTCCGTATGTNN
```

Section 14

```
                 (716) 716           730       740       750       760      770
Tap_BnaC.FAD2.a  (714) CGGTCTCTTCCGTTACGCCGCCGCGCAGGGAGTGGCCTCGATGGTCTGCTTCTAC
Cab_BnaC.FAD2.a  (714) CGGTCTCTTCCGTTACGCCGCCGCGCAGGGAGTGGCCTCGATGGTCTGCTTCTAC
Tap_BnaA.FAD2.a  (714) CGGTCTCTACCGCTACGCTGCTGTCCAAGGAGTTGCCTCGATGGTCTGCTTCTAC
Cab_BnaA.FAD2.a  (713) CGGTCTCTACCGCTACGCTGCTGTCCAAGGAGTTGCCTCGATGGTCTGCTTCTAC
Tap_BnaC.FAD2.b  (714) CGGTCTCTACCGCTACGCTGGTTCGCGAGGAGTGGCCTCGATGGTCTGTGTCTAC
Tap_BnaA.FAD2.b  (699) CGGTCTCTACCGTTACGCTGCTTCGCGAGGAGTAGCCTCTGTGGTCTGTGTCTAC
Cab_BnaA.FAD2.b  (699) NNNNNNNNNNNNNNNNNNNNNNNNGCGAGGAGTAGCCTCTGTGGTCTGTGTCTAC
```

Section 15

```
                 (771) 771      780       790       800       810           825
Tap_BnaC.FAD2.a  (769) GGAGTCCCGCTTCTGATTGTCAATGGTTTCCTCGTGTTGATCACTTACTTGCAGC
Cab_BnaC.FAD2.a  (769) GGAGTCCCGCTTCTGATTGTCAATGGTTTCCTCGTGTTGATCACTTACTTGCAGC
Tap_BnaA.FAD2.a  (769) GGAGTTCCTCTTCTGATTGTCAACGGGTTCTTAGTTTTGATCACTTACTTGCAGC
Cab_BnaA.FAD2.a  (768) GGAGTTCCTCTTCTGATTGTCAACGGGTTCTTAGTTTTGATCACTTACTTGCAGC
Tap_BnaC.FAD2.b  (769) GGAGTTCCGCTTATGATTGTCAACTGTTTCCTCGTCTTGATCACTTACTTGCAGC
Tap_BnaA.FAD2.b  (754) GGAGTTCCGCTTCTAATTGTCAACTGTTTCCTCGTCTTGATCACTTACTTGCAGC
Cab_BnaA.FAD2.b  (754) GGAGTTCCGCTTCTAATTGTCAACTGTTTCCTCGTCTTGATCACTTACTTGCAGC
```

Supplemental Figure 1. BnaFAD2 sequence alignment

```
                                                                     Section 16
                 (826)  826           840       850       860       870      880
Tap_BnaC.FAD2.a  (824)  ACACGCATCCTTCCCTGCCTCACTACGATTCGTCCGAGTGGGATTGGTTGAGGGG
Cab_BnaC.FAD2.a  (824)  ACACGCATCCTTCCCTGCCTCACTACGATTCGTCCGAGTGGGATTGGTTGAGGGG
Tap_BnaA.FAD2.a  (824)  ACACGCATCCTTCCCTGCCTCACTATGACTCGTCTGAGTGGGATTGGTTGAGGGG
Cab_BnaA.FAD2.a  (823)  ACACGCATCCTTCCCTGCCTCACTATGACTCGTCTGAGTGGGATTGGTTGAGGGG
Tap_BnaC.FAD2.b  (824)  ACACGCACCCTTCGCTGCCTCACTATGATTCTTCGGAGTGGGATTGGTTGAGAGG
Tap_BnaA.FAD2.b  (809)  ACACGCACCCTTCGCTGCCTCACTATGATTCTTCCGAGTGGGATTGGTTGAGAGG
Cab_BnaA.FAD2.b  (809)  ACACGCANNCTNCNCTGCCTCACTATGATTCTTCCGAGTGGGATTGGTTGAGAGG
                                                                     Section 17
                 (881)  881      890       900       910       920           935
Tap_BnaC.FAD2.a  (879)  AGCTTTGGCTACCGTTGACAGAGACTACGGAATCTTGAACAAGGTCTTCCACAAT
Cab_BnaC.FAD2.a  (879)  AGCTTTGGCTACCGTTGACAGAGACTACGGAATCTTGAACAAGGTCTTCCACAAT
Tap_BnaA.FAD2.a  (879)  AGCTTTGGCCACCGTTGACAGAGACTACGGAATCTTGAACAAGGTCTTCCACAAT
Cab_BnaA.FAD2.a  (878)  AGCTTTGGCCACCGTTGACAGAGACTACGGAATCTTGAACAAGGTCTTCCACAAT
Tap_BnaC.FAD2.b  (879)  AGCTTTGGCTACTGTGGATAGAGACTATGGAATCTTGAACAAGGTGTTTCATAAC
Tap_BnaA.FAD2.b  (864)  AGCTTTGGCTACTGTGGATAGAGACTATGGAATCTTGAACAAGGTGTTCCATAAC
Cab_BnaA.FAD2.b  (864)  AGCTTTGGCTACTGTGGATAGAGACTATGGAATCTTGAACAAGGTGTTCCATAAC
                                                                     Section 18
                 (936)  936           950       960       970       980      990
Tap_BnaC.FAD2.a  (934)  ATTACCGACACGCACGTGGCGCATCATCTGTTCTCCACGATGCCGCATTATCAC-
Cab_BnaC.FAD2.a  (934)  ATTACCGACACGCACGTGGCGCATCATCTGTTCTCCACGATGCCGCATTATCAC-
Tap_BnaA.FAD2.a  (934)  ATCACGGACACGCACGTGGCGCATCACCTGTTCTCGACCATGCCGCATTATCAT-
Cab_BnaA.FAD2.a  (933)  ATCACGGACACGCACGTGGCGCATCACCTGTTCTCGACCATGCCGCATTATCAT-
Tap_BnaC.FAD2.b  (934)  ATCACGGACACGCACGTGGCGCATCATCTGTTCTCGACGATGCCGCATTATAAC-
Tap_BnaA.FAD2.b  (919)  ATCACGGACACGCACGTGGCGCATCATCTGTTCTCGACGATGCCGCATTATAAC-
Cab_BnaA.FAD2.b  (919)  ATCACGGACACGCACGTGGCGCATCATCTGTTCTCCACGATGCCGCATTATAAC-
                                                                     Section 19
                 (991)  991      1000      1010      1020      1030         1045
Tap_BnaC.FAD2.a  (988)  GCGATGGAAGCTACCAAGGCGATAAAGCCGATACTG-GGAGAGTATTATCAGTTC
Cab_BnaC.FAD2.a  (988)  GCGATGGAAGCTACCAAGGCGATAAAGCCGATACTG-GGAGAGTATTATCAGTTC
Tap_BnaA.FAD2.a  (988)  GCGATGGAAGCTACGAAGGCGATAAAGCCGATACTG-GGAGAGTATTATCAGTTC
Cab_BnaA.FAD2.a  (987)  GCGATGGAAGCTACGAAGGCGATAAAGCCGATACTG-GGAGAGTATTATCAGTTC
Tap_BnaC.FAD2.b  (988)  GCGATGGAAGCGACCAAGGCGATAAAGCCGATACTT-GGAGAGTATTACCAGTTT
Tap_BnaA.FAD2.b  (973)  GCGATGGAAGCGACCAAGGCGATAAAGCCGATACTTTGGAGAGTATTACCAGTTT
Cab_BnaA.FAD2.b  (973)  GCGATGGAAGCGACCAAGGCGATAAAGCCGATACTTTGGAGAGTATTACCAGTTT
                                                                     Section 20
                (1046)  1046          1060      1070      1080      1090     1100
Tap_BnaC.FAD2.a (1042)  GATGGGACGCCGGTGGTTAAGGCGATGTGGAGGGAGGCGAAGGAGTGTATCTATG
Cab_BnaC.FAD2.a (1042)  GATGGGACGCCGGTGGTTAAGGCGATGTGGAGGGAGGCGAAGGAGTGTATCTATG
Tap_BnaA.FAD2.a (1042)  GATGGGACGCCGGTGGTTAAGGCGATGTGGAGGGAGGCGAAGGAGTGTATCTATG
Cab_BnaA.FAD2.a (1041)  GATGGGACGCCGGTGGTTAAGGCGATGTGGAGGGAGGCGAAGGAGTGTATCTATG
Tap_BnaC.FAD2.b (1042)  GATGGAACGCCGGTGGTTAAGGCGATGTGGAGGGAGGCGAAGGAGTGTATCTATG
Tap_BnaA.FAD2.b (1028)  GATGGAACGCCGGCGGTTAAGGCGATGTGGAGGGAGGCGAAGGAGTGTATCTATG
Cab_BnaA.FAD2.b (1028)  GATGGAACGCCGGCGGTTAAGGCGATGTGGAGGGAGGCGAAGGAGTGTATCTATG
```

Supplemental Figure 1. BnaFAD2 sequence alignment

Section 21

```
                 (1101) 1101     1110      1120      1130      1140          1155
Tap_BnaC.FAD2.a (1097) TGGAACCGGACAGGCAAGGTGAGAAGAAAGGTGTGTTCTGGTACAACAATAAGTT
Cab_BnaC.FAD2.a (1097) TGGAACCGGACAGGCAAGGTGAGAAGAAAGGTGTGTTCTGGTACAACAATAAGTT
Tap_BnaA.FAD2.a (1097) TGGAACCGGACAGGCAAGGTGAGAAGAAAGGTGTGTTCTGGTACAACAATAAGTT
Cab_BnaA.FAD2.a (1096) TGGAACCGGACAGGCAAGGTGAGAAGAAAGGTGTGTTCTGGTACAACAATAAGTT
Tap_BnaC.FAD2.b (1097) TTGAACCGGATAGGCAAGGTGAGAAGAAAGGTGTGTTCTGGTACAACAATAAGTT
Tap_BnaA.FAD2.b (1083) TTGAACCGGATAGGCAAGGTGAGAAGAAAGGTGTGTTCTGGTACAACAATAAGTT
Cab_BnaA.FAD2.b (1083) TTGAACCGGATAGGCAAGGTGAGAAGAAAGGTGTGTTCTGGTACAACAATAAGTT
```

Section 22

```
                 (1156) 1156 1159
Tap_BnaC.FAD2.a (1152) ATGA
Cab_BnaC.FAD2.a (1152) ATGA
Tap_BnaA.FAD2.a (1152) ATGA
Cab_BnaA.FAD2.a (1151) ATGA
Tap_BnaC.FAD2.b (1152) ATGA
Tap_BnaA.FAD2.b (1138) ATGA
Cab_BnaA.FAD2.b (1138) ATGA
```

Supplemental Figure 2. BnaFAD2 amino acid alignment

Section 1

```
                   (1) 1       10        20        30        40              56
Tap_BnaC.FAD2.a    (1) MGAGGRMQVSPPSKKSETDTIKRVPCETPPFTVGELKKAIPPHCFKRSIPRSFSYL
Cab_BnaC.FAD2.a    (1) MGAGGRMQVSPPSKKSETDTIKRVPCETPPFTVGELKKAIPPHCFKRSIPRSFSYL
Tap_BnaA.FAD2.a    (1) MGAGGRMQVSPPSKKSETDNIKRVPCETPPFTVGELKKAIPPHCFKRSIPRSFSYL
Cab_BnaA.FAD2.a    (1) MGAGGRMQVSPPSKKSETDNIKRVPCETPPFTVGELKKAIPPHCFKRSIPRSFSYL
Tap_BnaC.FAD2.b    (1) MGAGGRMQVSPPSSSPETKTLKRVPCETPPFTLGDLKKAIPPHCFKRSIPRSFSYL
Tap_BnaA.FAD2.b    (1) MGAGGRMQVSPPSSSPGTNTLKRVPCETPPFTLGDLKKAIPPHCFKRSIPRSFSSS
Cab_BnaA.FAD2.b    (1) MGAGGRMQVSPPSSSPGTNTLKRVPCETPPFTLGDLKKAIPPHCFKRSIPRSFSSS
```

Section 2

```
                  (57) 57           70        80        90        100        112
Tap_BnaC.FAD2.a   (57) IWDIIIASCFYYVATTYFPLLPHPLSYFAWPLYWACQGCVLTGVWVIAHECGHHAF
Cab_BnaC.FAD2.a   (57) IWDIIIASCFYYVATTYFPLLPHPLSYFAWPLYWACQGCVLTGVWVIAHECGHHAF
Tap_BnaA.FAD2.a   (57) IWDIIIASCFYYVATTYFPLLPHPLSYFAWPLYWACQGCVLTGVWVIAHECGHHAF
Cab_BnaA.FAD2.a   (57) IWDIIIASCFYYVATITSLSSLTLSPTSPGLSTGPARAASPASGSPTSAATTPSAT
Tap_BnaC.FAD2.b   (57) LFDILVSSSLYHLSTAYFPLLPHPLPYLAWPLYWACQGCVLTGLWVIAHECGHHAF
Tap_BnaA.FAD2.b   (57) TSSSPPRLLPLPPLHSLLPSPLPRLTPLLGLPRLRPNGPLGHSPRVRPPRLQRPPV
Cab_BnaA.FAD2.b   (57) TSSSPPRLLPLPPLHSLLPSPLPRLTPLLGLPRLRPNGPLGHSPRVRPPRLQRPPV
```

Section 3

```
                 (113) 113    120       130       140       150             168
Tap_BnaC.FAD2.a  (113) SDYQWLDDTVGLIFHSFLLVPYFSWKYSHRRHHSNTGSLERDEVFVPKKKSDIKWY
Cab_BnaC.FAD2.a  (113) SDYQWLDDTVGLIFHSFLLVPYFSWKYSHRRHHSNTGSLERDEVFVPKKKSDIKWY
Tap_BnaA.FAD2.a  (113) SDYQWLDDTVGLIFHSFLLVPYFSWKYSHRRHHSNTGSLERDEVFVPKKKSDIKWY
Cab_BnaA.FAD2.a  (113) TSGWTTPSASSSTPSSSSLTSPGS---TVIDATIPTLAPSRETKCLSPRRSQTSSG
Tap_BnaC.FAD2.b  (113) SDHQWLDDAVGLVFHSFLLVPYFSWKYSHRRHHSNTGSLERDEVFVPKKKSDIKWY
Tap_BnaA.FAD2.b  (113) AGRRRRPRLPLLPPRPVLLLEVHP------RHHSNTGSLDRDEVFVPKKKSDIKWY
Cab_BnaA.FAD2.b  (113) AGRRRRPRLPLLPPRPVLLLEVHP------RHHSNTGSLDRDEVFVPKKKSDIKWY
```

Section 4

```
                 (169) 169        180       190       200       210           224
Tap_BnaC.FAD2.a  (169) GKYLNNPLGRTVMLTVQFTLGWPLYLAFNVSGRPYDGGFACHFHPNAPIYNDRERL
Cab_BnaC.FAD2.a  (169) GKYLNNPLGRTVMLTVQFTLGWPLYLAFNVSGRPYDGGFACHFHPNAPIYNDRERL
Tap_BnaA.FAD2.a  (169) GKYLNNPLGRTVMLTVQFTLGWPLYLAFNVSGRPYDGGFACHFHPNAPIYNDRERL
Cab_BnaA.FAD2.a  (166) TASTSTTLWDAP-C-RFSSLSAGLCT-PSTSRGDLTTAASLAISTPTLPSTTTVSV
Tap_BnaC.FAD2.b  (169) GKYLNNPLGRTVMLTVQFTLGWPLYLAFNVSGRPYSDGFACHFHPNAPIYNDRERL
Tap_BnaA.FAD2.b  (163) GKYLNNPLGRTVMLTVQFKLGWPLYLAFNVSGRPYSDGFACHFHPNAPIYNDRERL
Cab_BnaA.FAD2.b  (163) GKYLNNPLGRTVMLTVQFKLGWPLYLAFNVSGRPYSDGFACHFHPNAPIYNDRERL
```

Section 5

```
                 (225) 225  230       240       250       260       270       280
Tap_BnaC.FAD2.a  (225) QIYISDAGILAVCYGLFRYAAA-----QGVASMVCFY---GVPLLIVNGFLVLITY
Cab_BnaC.FAD2.a  (225) QIYISDAGILAVCYGLFRYAAA-----QGVASMVCFY---GVPLLIVNGFLVLITY
Tap_BnaA.FAD2.a  (225) QIYISDAGILAVCYGLYRYAAV-----QGVASMVCFY---GVPLLIVNGFLVLITY
Cab_BnaA.FAD2.a  (219) SRYTSPTLASSPSATVSTATLLSKELPRWSASTEFLFLSTGS-F-SLTCSTRILPC
Tap_BnaC.FAD2.b  (225) QIYISDAGVLSVCYGLYRYAGS-----RGVASMVCVY---GVPLMIVNCFLVLITY
Tap_BnaA.FAD2.b  (219) QIYISDAGVLSVCYGLYRYAAS-----RGVASVVCVY---GVPLLIVNCFLVLITY
Cab_BnaA.FAD2.b  (219) QIYISDAGVLSVCXXXXXXXXX-----RGVASVVCVY---GVPLLIVNCFLVLITY
```

Supplemental Figure 2. BnaFAD2 amino acid alignment

```
                                                                          Section 6
                  (281) 281       290       300       310       320            336
Tap_BnaC.FAD2.a (273) LQHTHPSLPHYDSSEWDWLRGALATVDRDYGILNKVFHNITDTHVAHHLFSTMPHY
Cab_BnaC.FAD2.a (273) LQHTHPSLPHYDSSEWDWLRGALATVDRDYGILNKVFHNITDTHVAHHLFSTMPHY
Tap_BnaA.FAD2.a (273) LQHTHPSLPHYDSSEWDWLRGALATVDRDYGILNKVFHNITDTHVAHHLFSTMPHY
Cab_BnaA.FAD2.a (273) LTMTRLSGIG--GELWPPLTETTES-TRSSTISRTRTWRITCSRPCRIIMRWKLRR
Tap_BnaC.FAD2.b (273) LQHTHPSLPHYDSSEWDWLRGALATVDRDYGILNKVFHNITDTHVAHHLFSTMPHY
Tap_BnaA.FAD2.b (267) LQHTHPSLPHYDSSEWDWLRGALATVDRDYGILNKVFHNITDTHVAHHLFSTMPHY
Cab_BnaA.FAD2.b (267) LQHTXXXLPHYDSSEWDWLRGALATVDRDYGILNKVFHNITDTHVAHHLFSTMPHY
                                                                          Section 7
                  (337) 337          350       360       370       380         392
Tap_BnaC.FAD2.a (329) HAMEATKAIKPILGEYYQFDGTPVVKAMWREAKECIYVEPDR--QGEKKGVFWYNN
Cab_BnaC.FAD2.a (329) HAMEATKAIKPILGEYYQFDGTPVVKAMWREAKECIYVEPDR--QGEKKGVFWYNN
Tap_BnaA.FAD2.a (329) HAMEATKAIKPILGEYYQFDGTPVVKAMWREAKECIYVEPDR--QGEKKGVFWYNN
Cab_BnaA.FAD2.a (326) R-SRYWESIISSMGRRWLR---RCGG---RRRSVSMWNRTGKVRRKVCSGTTISY-
Tap_BnaC.FAD2.b (329) NAMEATKAIKPILGEYYQFDGTPVVKAMWREAKECIYVEPDR--QGEKKGVFWYNN
Tap_BnaA.FAD2.b (323) NAMEATKAIKPILWRVLPV--WNAGG-GDVEGGEGVYLC-TG-AR-EERCVLVQQ-
Cab_BnaA.FAD2.b (323) NAMEATKAIKPILWRVLPV--WNAGG-GDVEGGEGVYLC-TG-AR-EERCVLVQQ-
                                                                          Section 8
                  (393) 393 394
Tap_BnaC.FAD2.a (383) KL
Cab_BnaC.FAD2.a (383) KL
Tap_BnaA.FAD2.a (383) KL
Cab_BnaA.FAD2.a (374) --
Tap_BnaC.FAD2.b (383) KL
Tap_BnaA.FAD2.b (372) VM
Cab_BnaA.FAD2.b (372) VM
```

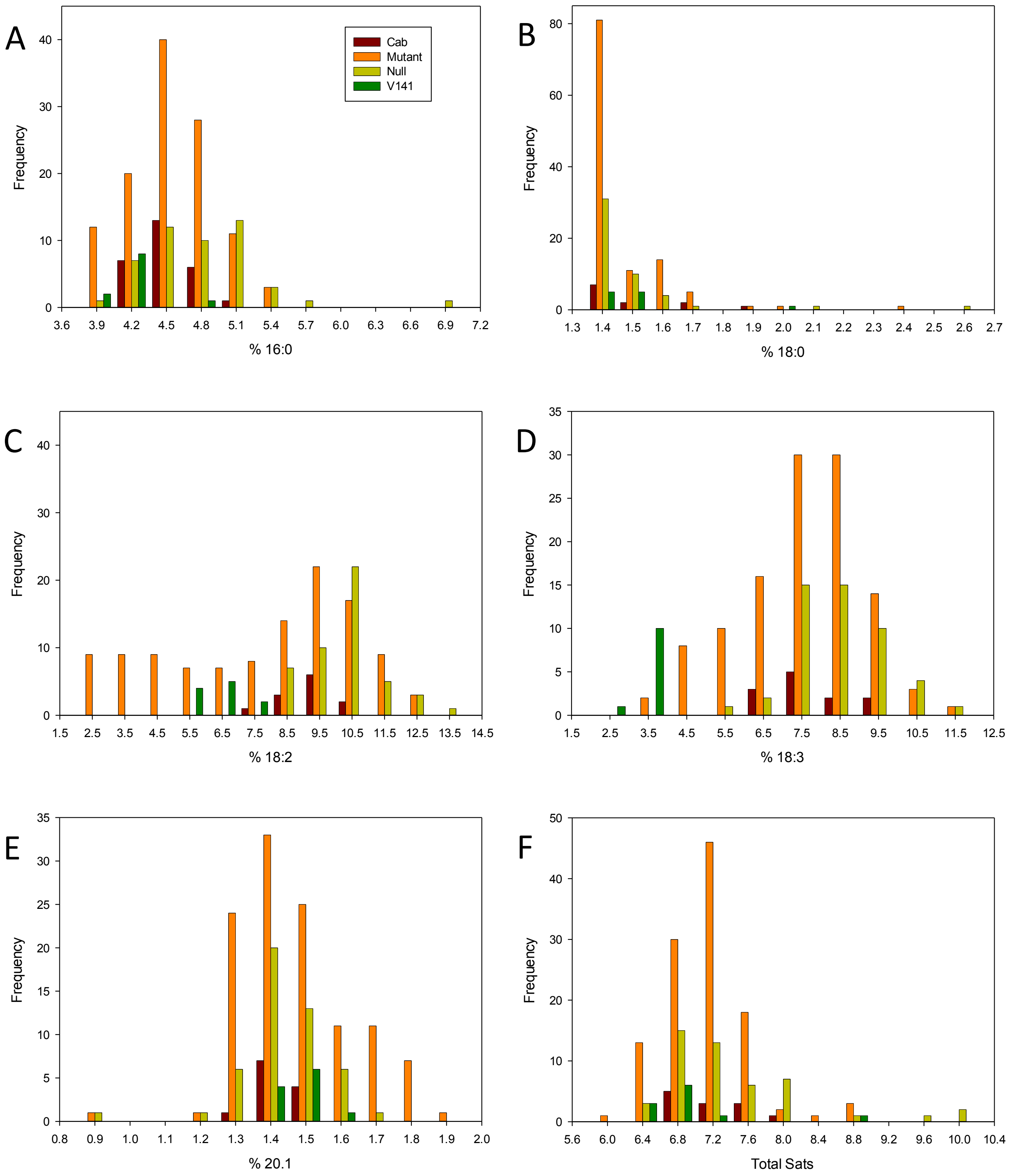

Supplemental Figure 3. Distribution of fatty acid composition of seeds in mutant and control lines. A) Palmitic acid (C16:0), B) Stearic acid (C18:0), C) Linoleic acid (C18:2), D) Linolenic acid (C18:3), E) 9-Eicosenoic acid (C20:1) and F) total saturated fatty acids in seeds of *B. napus* variety Cabriolet (Cab), *BnaA.FAD2.a* mutated lines (Mutant), outsegregant lines (Null) and a commercial high oleic, low linolenic line (V141).

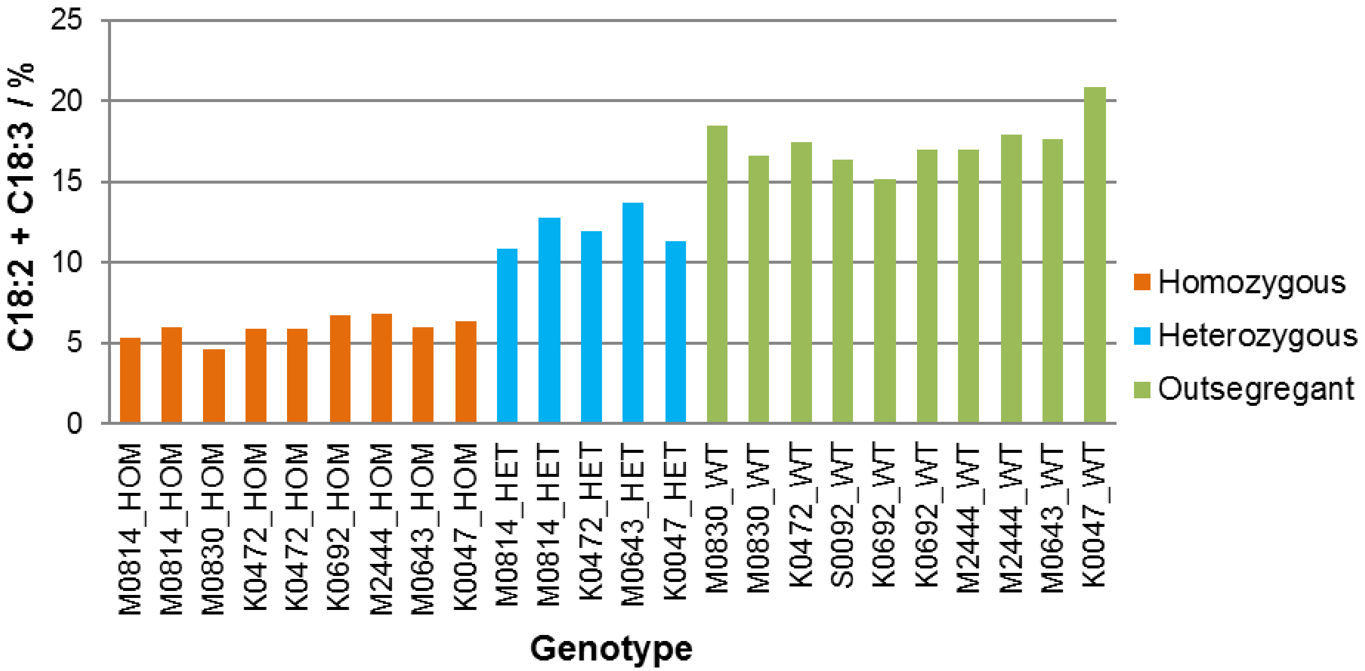

Supplemental Figure 4. Polyunsaturated fatty acid content of seeds from plants segregating for mutations *in BnaC.FAD2.a* that have major effects on oil composition
